# Paracrine signalling between intestinal epithelial and tumour cells induces a regenerative programme

**DOI:** 10.7554/eLife.76541

**Published:** 2022-05-11

**Authors:** Guillaume Jacquemin, Annabelle Wurmser, Mathilde Huyghe, Wenjie Sun, Zeinab Homayed, Candice Merle, Meghan Perkins, Fairouz Qasrawi, Sophie Richon, Florent Dingli, Guillaume Arras, Damarys Loew, Danijela Vignjevic, Julie Pannequin, Silvia Fre

**Affiliations:** 1 Institut Curie, Laboratory of Genetics and Developmental Biology, PSL Research University, INSERM U934, CNRS UMR3215 Paris France; 2 Sorbonne University, UPMC University of Paris VI Paris France; 3 IGF, University of Montpellier, CNRS, INSERM Montpellier France; 4 Institut Curie, PSL Research University, CNRS UMR 144 Paris France; 5 Institut Curie, PSL Research University, Laboratory of Mass Spectrometry and Proteomics Paris France; Brown University United States; Brown University United States

**Keywords:** organoids, colon cancer, YAP signalling, Mouse

## Abstract

Tumours are complex ecosystems composed of different types of cells that communicate and influence each other. While the critical role of stromal cells in affecting tumour growth is well established, the impact of mutant cancer cells on healthy surrounding tissues remains poorly defined. Here, using mouse intestinal organoids, we uncover a paracrine mechanism by which intestinal cancer cells reactivate foetal and regenerative YAP-associated transcriptional programmes in neighbouring wildtype epithelial cells, rendering them adapted to thrive in the tumour context. We identify the glycoprotein thrombospondin-1 (THBS1) as the essential factor that mediates non-cell-autonomous morphological and transcriptional responses. Importantly, Thbs1 is associated with bad prognosis in several human cancers. This study reveals the THBS1-YAP axis as the mechanistic link mediating paracrine interactions between epithelial cells in intestinal tumours.

## Introduction

It is now well established that tumour formation and progression are vastly influenced by the crosstalk between cancer cells and their environment, involving complex remodelling of the extracellular matrix and interaction with stromal cells, such as cancer-associated fibroblasts, myofibroblasts, pericytes, vascular and lymphatic endothelial cells, as well as different types of inflammatory immune cells (Marusyk et al., 2014; Cleary et al., 2014; Hanahan and Coussens, 2012). Remodelling of the tumour microenvironment has been shown to support tumour growth through neo-angiogenesis as well as via direct effects on cancer cells exposed to pro-inflammatory and pro-survival cytokines (Balkwill et al., 2012). However, paracrine interactions have mostly been studied among different cell types and little is known about communication between cancer and adjacent normal epithelial cells that could contribute to tumour formation and progression. Defining the mechanisms allowing paracrine interactions between tumour and normal epithelial cells requires an understanding of how different cells persist and expand within a tumour and is crucial to dissect intratumoral heterogeneity. It is noteworthy that these questions have lately received a special attention, and several studies addressing the coexistence and complex relationship between mutant and wildtype (WT) epithelial cells in the context of intestinal tumours have been published over the past year (Yum et al., 2021; Flanagan et al., 2021; van Neerven et al., 2021; Krotenberg Garcia et al., 2021).

We have recently reported that intestinal stem cells can be found within intestinal tumours and contribute to tumour growth (Mourao et al., 2019), suggesting the existence of bidirectional communications between tumour and normal epithelial cells. A recent study has also provided evidence that a parenchymal response of normal epithelial cells favours tumour growth and dissemination (Ombrato et al., 2019). The advent of 3D organotypic cultures able to faithfully recapitulate the morphology and physiology of intestinal cells in a mesenchyme-free environment has now allowed us to address the unresolved question of epithelial-specific interactions in the context of intestinal tumoroids.

Intestinal organoids are well-characterised stem-cell-derived structures (Sato and Clevers, 2013). Importantly, organoids generated from normal mouse intestinal crypts consistently present a stereotypical ‘budding’ morphology, with proliferative crypts (or buds) and a terminally differentiated villus domain. On the other hand, cells derived from *Apc* mutant intestinal tumours generally grow as hyperproliferative and non-polarised hollow spheres or cysts (Drost et al., 2015; Sato et al., 2011; Jardé et al., 2013; Schwank et al., 2013; Germann et al., 2014; Onuma et al., 2013).

To study epithelial communications in a stroma-free environment, we analysed the influence of mutant organoids derived from primary mouse tumours (hereafter defined as ‘tumoroids’) on WT small intestinal organoids. We discovered that the co-culture of tumoroids and budding organoids quickly induced a hyperproliferative cystic morphology (referred to as ‘cysts’ hereafter) in a fraction of WT organoids. This interaction did not require cell contact as the effect was recapitulated by the conditioned medium (cM) from tumoroids. We found that the secreted glycoprotein thrombospondin-1 (THBS1) was responsible for mediating these paracrine communications through Yap pathway activation. Under the influence of tumour-derived THBS1, WT cells activate the YAP signalling pathway and induce foetal and regenerative transcriptional programmes, which cause their hyperproliferation and failure to properly differentiate. Importantly, we show that the THBS1/YAP1 signalling axis we discovered in organoids is conserved in both mouse and human colon cancer and propose that this early mechanism of non-cell-autonomous epithelial communication is critical for the establishment of a primary tumour. Of medical relevance, we also found that THBS1 expression is necessary for tumoroids’ growth. These studies offer novel insights into the molecular mechanisms responsible for tumour establishment and provide an attractive therapeutic avenue in targeting THBS1 to reduce tumour complexity and heterogeneity.

## Results

### Tumour cells induce a cancer-like behaviour in WT intestinal epithelial cells

**Figure 1.**
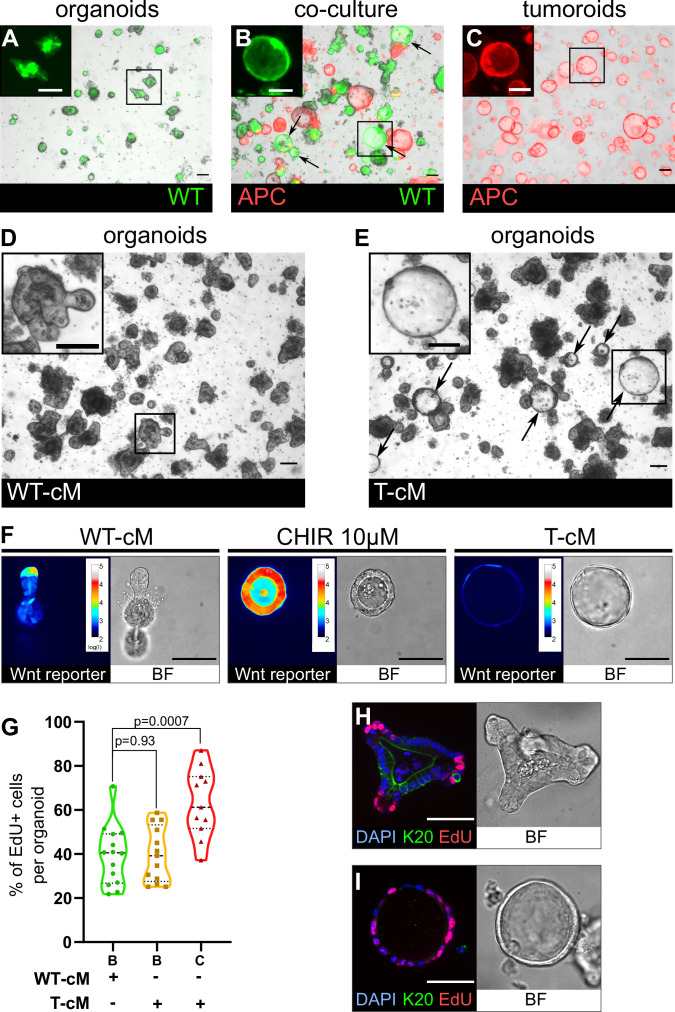
Tumoroids secrete soluble factors that induce a tumour-like cystic morphology in wildtype (WT) organoids. (**A**) WT budding organoids marked by LifeAct-GFP (in green) after 24 hr in culture in organoid medium (ENR). (**B**) WT organoids marked by LifeAct-GFP after 24 hr in co-culture with tdTomato-expressing tumoroids in ENR. Arrows indicate WT (green) cystic organoids. (**C**) APC mutant cystic tumoroids marked by tdTomato after 24 hr in culture. (**D, E**) WT budding organoids cultured for 24 hr in conditioned medium from WT organoids (WT-cM in **D**) or tumoroids (T-cM in **E**). Arrows indicate WT cystic organoids in (**E**). (**F**) Representative images of WT organoids expressing the Wnt reporter 7TG exposed to WT-cM, 10 µM CHIR99021 (CHIR 10 µM) or T-cM for 24 hr. Pseudo-colour shows log10 intensities of the reporter fluorescence. (**G**) Quantification of the percentage of EdU+ cells per organoid for budding organoids grown in WT-cM (B – WT-cM, n = 14), budding organoids grown in T-cM (B – T-cM, n = 13), or cystic organoids grown in T-cM (C – T-cM, n = 9). (**H, I**) Immunofluorescence for proliferative cells (EdU in red) and differentiated cells (anti-Keratin 20 in green) in WT organoids grown in WT-cM (**H**) or T-cM (**I**) for 24 hr. The corresponding bright-field (BF) images are shown in the right panels. DAPI stains DNA in blue. Scale bar = 100 µm. Statistical analysis was performed with two-tailed unpaired Welch’s *t*-tests. Figure 1—source data 1.Source data related to Figure 1G.

To mimic intratumoral heterogeneity in stroma-free conditions, we co-cultured tumoroids derived from primary intestinal tumours of *Apc*^1638N/+^ mutant mice (Fodde et al., 1994), labelled by membrane tdTomato (Muzumdar et al., 2007) with WT GFP-marked small intestinal organoids, derived from LifeAct-GFP mice (Riedl et al., 2008; Figure 1—figure supplement 1A). Within 24–48 hr of co-culture with tumoroids, WT organoids (up to 20%), normally displaying the stereotypical budding morphology (Sato et al., 2009; Figure 1A), adopted an unpolarised hollow cystic shape presenting a diameter larger than 100 µm (indicated by arrows in Figure 1B), closely resembling the morphology of *Apc* mutant tumoroids (Schwank et al., 2013; Figure 1C). To assess if this morphological change was due to mid-range paracrine or juxtacrine signals, we cultured WT organoids in cM from either wildtype (WT-cM) or tumour (T-cM) organoids. Consistent with our observations from co-cultures, WT organoids exposed to T-cM (Figure 1E), but not to WT-cM (Figure 1D), grew as cysts, suggesting that tumoroids secrete factors able to morphologically alter WT epithelial cells. Tumoroids derived from different primary tumours reproducibly induced the cystic ‘transformation’, albeit to variable extents (Figure 1—figure supplement 1B). Of interest, the cM from *Apc*^-/-^ organoids (derived from *Villin*^CreERT2^; *Apc*^flox/flox^ mice), where *Apc* knockout was induced by Cre recombination and did not rely on spontaneous *Apc* LOH, reproduced the effect of T-cM and induced a cystic morphology in WT organoids, confirming a direct effect caused by aberrant Wnt signalling (Figure 1—figure supplement 1C). The morphological change was all the more remarkable as it occurred within 6–12 hr of exposure to T-cM (Figure 1—figure supplement 1D and E) and was reversed after 2–4 days, if no fresh medium was added, suggesting exhaustion of the responsible factor(s) and ruling out the possibility of acquired genetic mutations in normal organoids. Since a cystic organoid morphology has been linked to Wnt pathway activation, we analysed the expression of the quantitative Wnt reporter 7TG (Brugmann et al., 2007). As shown in Figure 1F, cystic organoids grown in T-cM did not present canonical Wnt pathway activation (Figure 1F, right panel), unlike organoids stimulated with the small-molecule CHIR99021, an inhibitor of the enzyme GSK-3, widely used to simulate Wnt activation (Ring et al., 2003; Figure 1F, middle panel). Confirming these observations, we could not observe any significant difference in the number of Lgr5+ cells in the presence of T-cM compared to both WT-cM and normal ENR (Figure 1—figure supplement 1F), whereas exposure to the GSK3 inhibitor CHIR99021 (ENRC) resulted, as expected, in a significant increase in GFP+ cells. Despite absence of Wnt activation and similar numbers of Lgr5-expressing cells (Figure 1F, Figure 1—figure supplement 1F), we found that T-cM-exposed cystic organoids presented a higher proportion of cycling cells (compare cystic ‘C’ and budding ‘B’ in Figure 1G and Figure 1—figure supplement 1G). Moreover, while proliferative cells were restricted to the crypts in control organoids exposed to WT-cM (Figure 1H), cystic organoids in T-cM displayed undifferentiated proliferative cells scattered throughout the newly formed cysts (Figure 1I). The increase in proliferative cells is linked to defective enterocyte differentiation, as shown by loss of Keratin 20 expression (Figure 1I).

**Figure 1—figure supplement 1.**
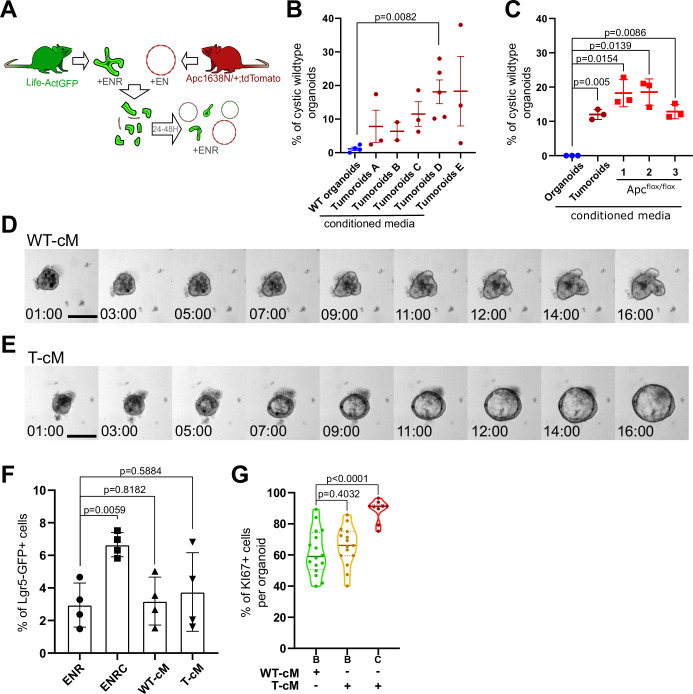
Related to Figure 1. (**A**) Experimental design of co-culture experiments with green organoids and red tumoroids at 3:1 ratio (wildtype [WT] organoids:tumoroids). ENR: medium containing EGF, Noggin, and R-spondin1. (**B**) Quantification of the percentage of WT cystic organoids in the presence of conditioned medium (cM) derived from the indicated cultures. (**C**) Quantification of the percentage of WT cystic organoids in the presence of cM derived from WT organoids, tumoroids, or three different *Apc*^-/-^ organoids generated from hyperplastic small intestine of *Villin*^CreERT2^;*Apc*^flox/flox^ mice. (**D, E**) Time-lapse analysis of the growth of WT organoids in the presence of WT-cM (**D**) or T-cM (**E**) (in hours:minutes). (**F**) Quantification of the percentage of Lgr5-GFP+ cells in WT organoids treated with ENR, ENR+CHIR99021 (ENRC), WT-cM or T-cM (n = 4 for each condition). (**G**) Quantification of the percentage of Ki67+ proliferative cells per organoid for budding organoids grown in WT-cM (B – WT-cM, n = 16), budding organoids grown in T-cM (B – TcM, n = 15) or cystic organoids grown in T-cM (C – T-cM, n = 9). Scale bar = 100 µm. Graphs indicate average values ± SD. Statistical analysis was performed with two-tailed unpaired Welch’s *t*-tests. Figure 1—figure supplement 1—source data 1.Source data related to Figure 1—figure supplement 1B. Figure 1—figure supplement 1—source data 2.Source data related to Figure 1—figure supplement 1C. Figure 1—figure supplement 1—source data 3.Source data related to Figure 1—figure supplement 1F. Figure 1—figure supplement 1—source data 4.Source data related to Figure 1—figure supplement 1G.

### THBS1 mediates the organoid morphological and behavioural change

**Figure 2.**
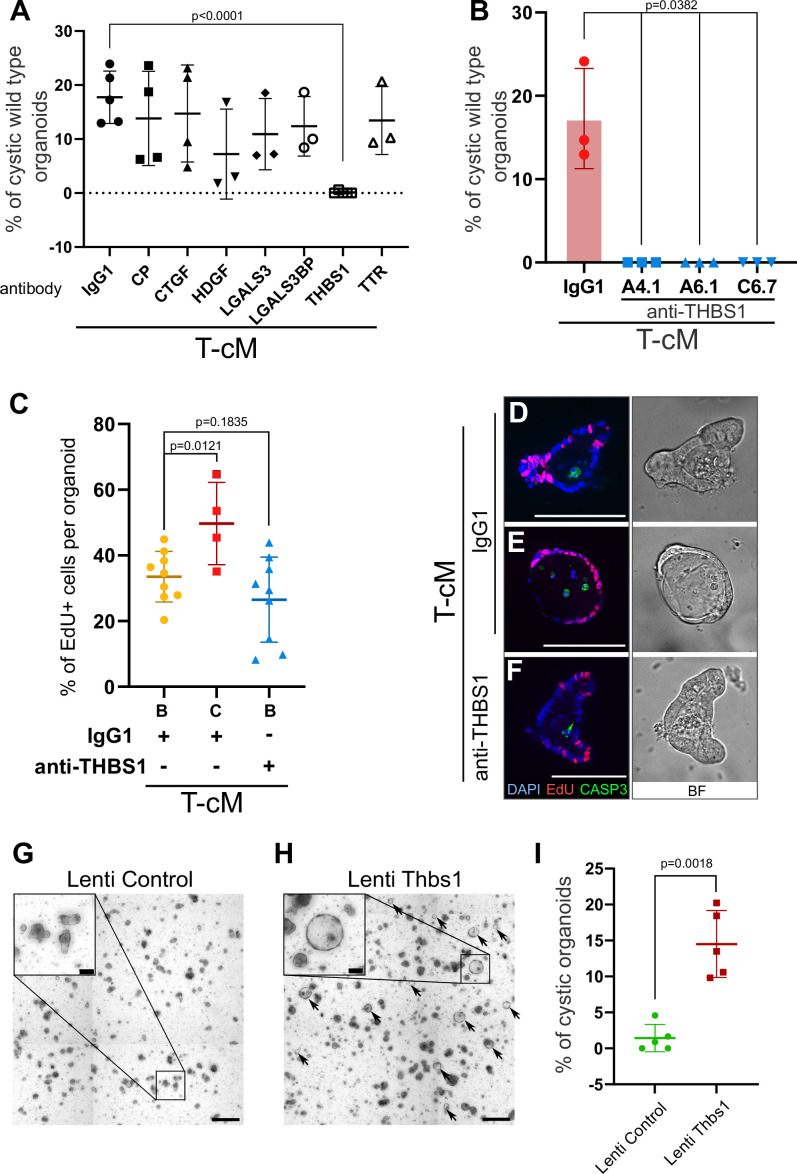
Thrombospondin-1 (THBS1) is necessary and sufficient for the morphological ‘transformation’ of wildtype (WT) organoids. (**A**) Quantification of the percentage of WT cystic organoids in T-cM upon neutralisation with blocking antibodies against ceruloplasmin (CP), connective tissue growth factor (CTGF), hepatoma-derived growth factor (HDGF), galectin-3 (LGALS3), galectin-3 binding protein (LGALS3BP), thrombospondin-1 (THBS1), and transthyretin (TTR) (5 µg/ml). (**B**) Quantification of the percentage of WT cystic organoids in T-cM upon neutralisation with three different blocking antibodies against THBS1 (clones A4.1, A6.1, and C6.7 at 5 µg/ml). (**C**) Quantification of the percentage of EdU+ cells (2 hr pulse) per organoid for WT budding (B – IgG1, n = 9) or cystic organoids (C – IgG1, n = 4) exposed to T-cM in the presence of IgG1 or antibodies anti-THBS1 (B – anti-THBS1, n = 9). (**D–F**) Whole-mount immunostaining for proliferation (EdU in red) and apoptosis (anti-cleaved caspase-3, CASP3 in green) of WT organoids exposed to T-cM with anti-IgG1 control (**D, E**) or anti-THBS1 (**F**) antibodies. DAPI stains DNA in blue. The corresponding bright-field (BF) images are shown on the right panels. (**G, H**) Representative pictures of self-transformed WT organoids overexpressing Thbs1 (Lenti-Thbs1 in H) and control organoids infected with an empty vector (Lenti-Control in **G**). Black arrows indicate cystic organoids. (**I**) Quantification of the percentage of cystic organoids in Thbs1-expressing cultures (Lenti-Thbs1) versus control cultures (Lenti-Control) grown for 24 hr in ENR medium (n = 5). Scale bars = 100 µm in (**D–F**) and in the insets of (**G, H**) and 500 µm in (**G, H**) low magnification. Graphs indicate average values ± SD. Statistical analysis was performed with two-tailed unpaired Welch’s *t*-tests. Figure 2—source data 1.Source data related to Figure 2A. Figure 2—source data 2.Source data related to Figure 2B. Figure 2—source data 3.Source data related to Figure 2C. Figure 2—source data 4.Source data related to Figure 2I.

Cancer cells are known to secrete numerous factors to remodel the tumour microenvironment. Having established that the morphological change was mediated by proteins present in the T-cM, since the effect was abolished upon proteinase K treatment (Figure 2—figure supplement 1A), we performed a quantitative proteomics analysis by Stable Isotope Labelled Amino acids in Culture (SILAC) mass spectrometry to define the composition of the T-cM relative to WT-cM. Gene Ontology (GO) analysis of the proteins over-represented in T-cM showed enrichment in cell adhesion, wound healing, and generally ECM-related GO terms (Figure 2—figure supplement 1B). We selected secreted factors that were enriched in T-cM compared to WT-cM (Figure 2—figure supplement 1C) and explored their possible involvement in the morphological ‘transformation’ using neutralising antibodies. Among the tested candidates, we found that neutralisation of the secreted glycoprotein THBS1 alone was sufficient to entirely abolish the morphological change of WT organoids after 24 hr (Figure 2A, Figure 2—figure supplement 1D). Neutralisation of Thbs1 with three blocking antibodies, targeting different epitopes of the protein to exclude any potential non-specific binding, was sufficient to completely block the cystic morphology (Figure 2B, Figure 2—figure supplement 1E). These experiments demonstrated that THBS1 was necessary for the observed cystic phenotype.

**Figure 2—figure supplement 1.**
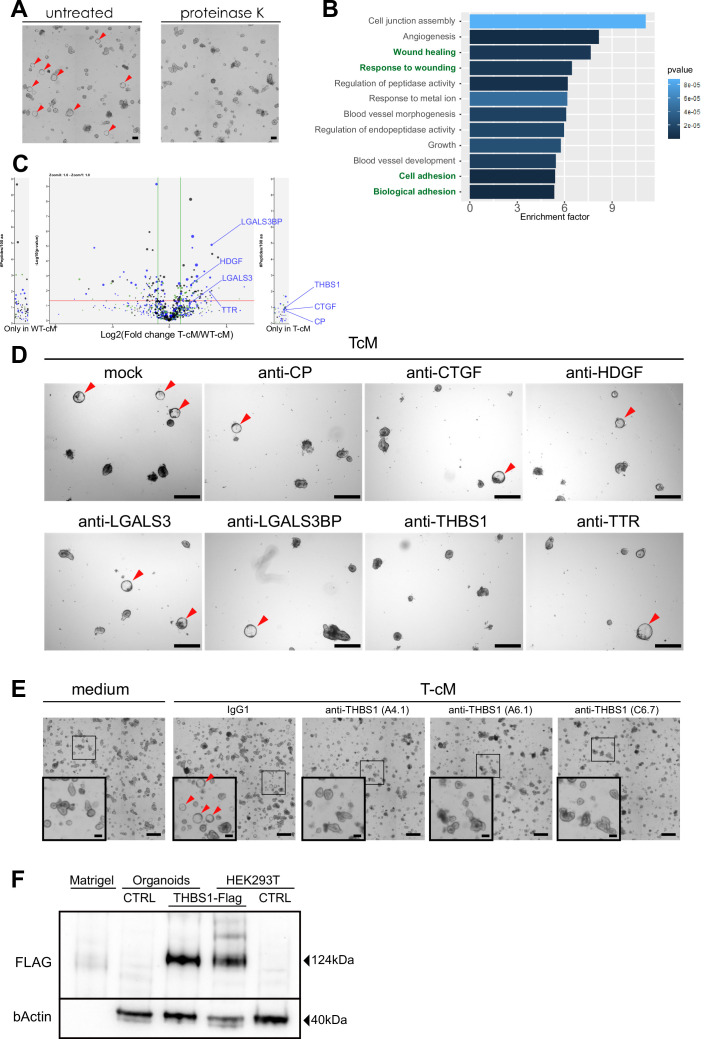
Related to Figure 2. (**A**) Proteinase K treatment of tumour conditioned medium (T-cM) abolished the cystic transformation. (**B**) Gene Ontology (GO) analysis of significantly enriched or unique proteins in T-cM compared to wildtype conditioned medium (WT-cM). (**C**) Volcano plot showing differential enrichment of proteins in T-cM compared to WT-cM by Stable Isotope Labelled Amino acids in Culture (SILAC)-based mass spectrometry. The selected candidates are indicated for one of the replicates in blue. The three different colours (black, blue, and green dots) correspond to three replicates (n = 3). (**D**) Representative bright-field images of WT organoids grown in T-cM upon neutralisation with blocking antibodies against the indicated proteins. (**E**) Representative bright-field images of WT organoids grown in normal medium (left panel) or T-cM upon neutralisation with control IgG1 or three different blocking antibodies against THBS1 (clones A4.1, A6.1, and C6.7). (**F**) Western blot anti-FLAG (targeting overexpressed THBS1-FLAG) and β-actin (loading control) in pure Matrigel, WT organoids infected with a control lentivirus (CTRL) or with lenti-Thbs1 (THBS1-Flag), HEK293T cells infected with lenti-Thbs1 (THBS1-Flag) or control lentivirus (CTRL). Scale bar = 100 µm in (**A**), 500 µm in (**D, E**) (100 µm in insets in **E**). Cystic organoids are indicated by red arrowheads in (**A**), (**D**), and (**E**). Figure 2—figure supplement 1—source data 1.Original image of the complete gel for the Western blot presented in Figure 2—figure supplement 1F. Figure 2—figure supplement 1—source data 2.Original image of the complete gel for the Western blot presented in Figure 2—figure supplement 1F.

In order to test if THBS1 neutralisation was also able to rescue the ectopic proliferation, we counted the number of proliferative cells per organoid. Consistent with our previous observations, cystic organoids presented ectopically proliferating cells when cultured with IgG1 control antibodies (Figure 2C and E). However, addition of anti-THBS1 antibodies abolished ectopic proliferation and restricted EdU+ cells exclusively to the crypts (Figure 2C and F). Treatment with anti-THBS1 antibodies did not present toxicity to normal organoids, as no increase in apoptotic cells was observed (Figure 2D–F). Moreover, THBS1 was sufficient to induce the morphological change, since its ectopic expression in WT organoids (Figure 2—figure supplement 1F) also led to cyst development (Figure 2G–I), to the same extent as T-cM (Figure 2I). Of note, culture of WT organoids in the presence of recombinant THBS1 did not elicit any effect, possibly due to the lack of essential post-translational modifications.

### THBS1 is necessary for the growth of tumoroids but not of normal organoids

**Figure 3.**
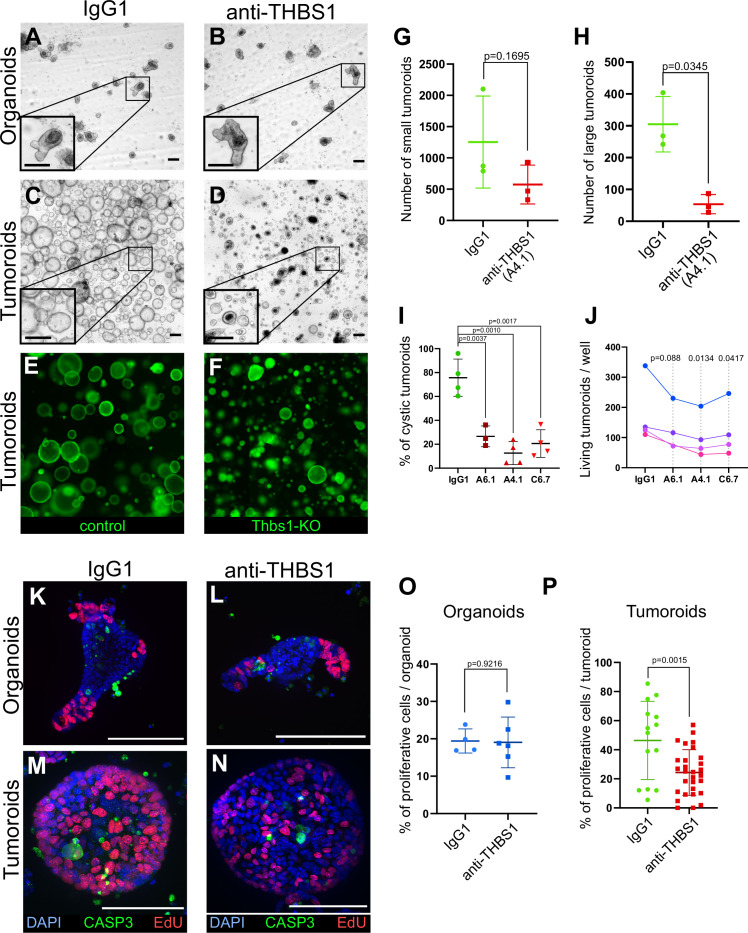
Thrombospondin-1 (THBS1) is essential for the growth of tumoroids. (**A–D**) Representative bright-field images of wildtype (WT) organoids (**A, B**) or tumoroids (**C, D**) incubated with IgG1 isotype control antibodies (**A, C**) or anti-THBS1 A6.1-neutralising antibody (**B, D**) (10 µg/ml). (**E, F**) Representative images of tumoroids infected with a lentivirus CRISPR-GFP without sgRNA (control in **E**) or with an sgRNA targeting Thbs1 (Thbs1-KO in **F**) 48 hr after replacement of single-cell seeding medium (ENRC) by tumoroid medium (EN). (**G, H**) Quantification of the number of tumoroids upon antibody neutralisation relative to their size: small tumoroids between 30 and 150 µm in (**G**); large tumoroids of more than 150 µm diameter in (**H**). (**I**) Quantification of the percentage of cystic tumoroids upon treatment by IgG1 isotype control antibodies or three different neutralising antibodies targeting THBS1 (as indicated) for 48 hr. (**J**) Paired quantification of the number of living tumoroids derived from four independent tumours (from four mice) upon treatment with three neutralising antibodies targeting THBS1 for 48 hr. Antibody concentration: 10 µg/ml. (**K–N**) Immunofluorescence staining for proliferative cells (EdU in red) and apoptosis (anti-cleaved caspase-3, CASP3 in green) in WT organoids (**K, L**) or tumoroids (**M, N**) exposed to IgG1 isotype control antibodies (**K, M**) or to anti-THBS1 A6.1-neutralising antibody (**L, N**). (**O, P**) Quantification of EdU+ cells per organoid (**O**) or tumoroid (**P**) in the presence of IgG1 control or anti-THBS1 (A6.1) antibodies. Scale bars = 100 µm. Graphs indicate average values ± SD. Statistical analysis was performed with paired Student’s *t*-test in (**G**–**J**) and two-tailed unpaired Welch’s *t*-tests in (**O**) and (**P**). Figure 3—source data 1.Source data related to Figure 3G. Figure 3—source data 2.Source data related to Figure 3H. Figure 3—source data 3.Source data related to Figure 3I. Figure 3—source data 4.Source data related to Figure 3J. Figure 3—source data 5.Source data related to Figure 3O. Figure 3—source data 6.Source data related to Figure 3P.

We observed that Thbs1 is exclusively expressed by tumour but not WT intestinal cells (Figure 2—figure supplement 1C, Figure 5—figure supplement 1D); surprisingly, we found that THBS1 is also essential for tumoroids’ growth. Indeed, neutralisation of THBS1 for 48 hr specifically reduced tumoroid survival and considerably arrested their growth (Figure 3A–D and G–J, Figure 3—figure supplement 1A). Importantly, the same tumoroid growth inhibition was observed upon *Thbs1* genetic deletion (Figure 3E and F) using CRISPR-Cas9 knockout (Figure 3—figure supplement 1B–E). Quantification of the proportion of dividing cells showed that neutralisation of THBS1 significantly reduced tumoroids’ proliferative capacity (Figure 3M, N and P), without affecting the growth of normal organoids (Figure 3K, L and O), suggesting a promising therapeutic avenue.

**Figure 3—figure supplement 1.**
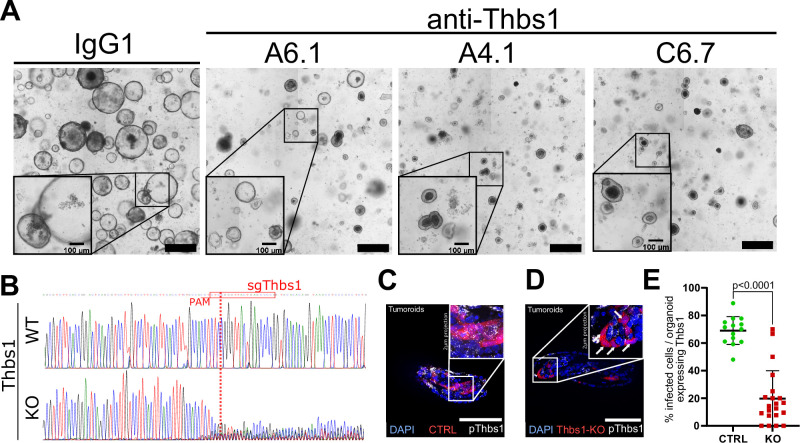
Related to Figure 3. (**A**) Representative images of tumoroids cultures grown for 48 hr in EN medium in the presence of control IgG1 or anti-THBS1-neutralising antibodies A6.1, A4.1, C6.7, as indicated. (**B**) Analysis of CRISPR edits from Sanger sequencing of DNA at the expected cut site for Cas9 in wildtype (WT) or THBS1 KO mouse embryonic fibroblasts infected with no sgRNA or sgRNA targeting Thbs1, respectively. Red boxes indicate the sgRNA sequences with the PAM sequence underlined in red; red vertical dashed lines indicate the expected cut site. (**C, D**) Representative images of tumoroids infected with lenti-CRISPR expressing no sgRNA (CTRL in **C**) or sgThbs1 (KO in **D**) probed for Thbs1 expression using single-molecule RNA fluorescence in situ hybridisation (smRNA FISH) with a probe recognising Thbs1 (pThbs1 in white). Red fluorescence indicates infected cells and DAPI stains the nuclei. (**E**) Quantification of the percentage of infected cells (red) expressing Thbs1 per organoid (n = 3). Scale bar = 500 µm in (**A**) and 100 µm in (**C, D**) and insets in (**A**). Statistical analysis was performed with two-tailed unpaired Welch’s *t*-tests. Figure 3—figure supplement 1—source data 1.Source data related to Figure 3—figure supplement 1E.

### WT organoids activate a regenerative/foetal transcriptional programme upon the influence of tumoroids’ conditioned medium

**Figure 4.**
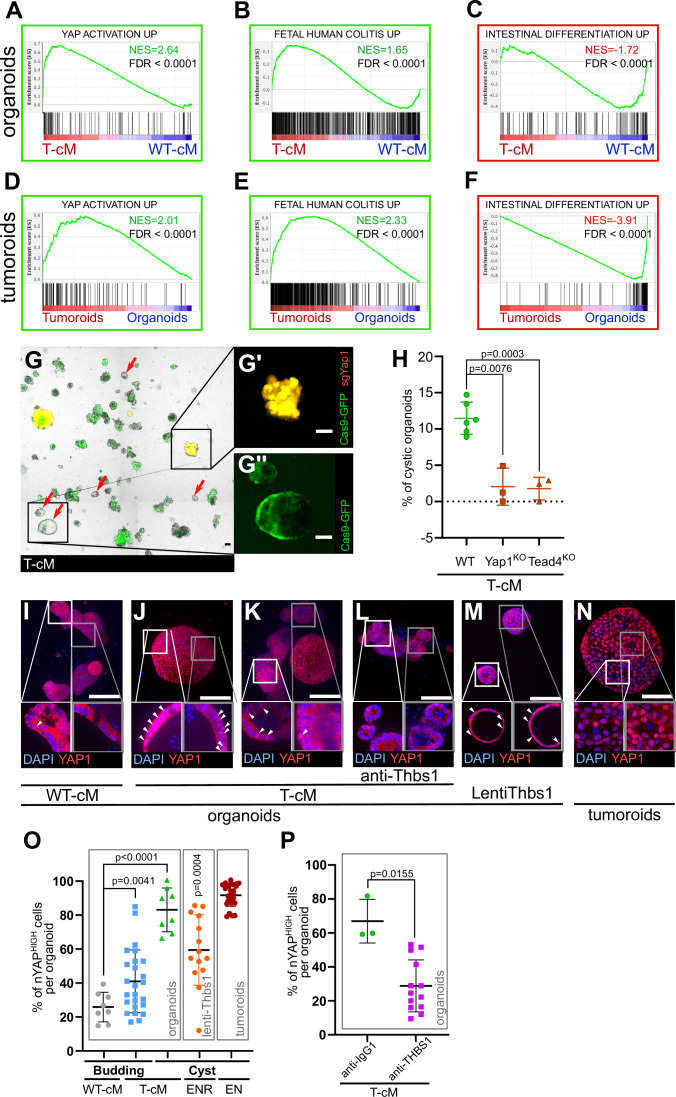
Tumour conditioned medium (T-cM) induces YAP pathway activation and a foetal-like state in wildtype (WT) organoids. Gene Set Enrichment Analyses (GSEA) showing the correlation between differentially expressed genes in WT organoids cultured in T-cM (**A–C**) or tumoroids (**D–F**) and the indicated transcriptional signatures. NES: Normalised Enrichment Score; green NES: positive correlation; red NES: inverse correlation. (**G**) Representative image of WT organoids expressing Cas9-GFP (in green) transduced with an sgRNA targeting Yap1 (sgYap1 in red). Higher magnification of a budding Yap1^KO^ organoid (in yellow in **G′**) and a cystic Yap1^WT^ organoid expressing only Cas9-GFP but no sgRNA (in green in **G′′**). (**H**) Percentage of cystic organoids induced by exposure to T-cM in WT, Yap1^KO^ or Tead4^KO^ organoids, as indicated. (**I–N**) Max projections of immunostaining for YAP1 (in red) of WT organoids exposed to WT-cM (**I**), or T-cM (**J–L**) for 24 hr presenting cystic (**J**) or budding (**K, L**) morphologies. Organoids in (**L**) are treated by neutralising antibodies targeting THBS1 (A6.1), which rescues the budding morphology. Organoids in (**M**) overexpress THBS1 (LentiThbs1) and tumoroids are shown in (**N**). DAPI stains DNA in blue. White arrowheads pinpoint YAP^HIGH^ cells in Z-section insets. (**O**) Quantification of the percentage of nuclear YAP (nYAP^HIGH^) cells/organoid based on the ratio of nuclear vs. cytoplasmic YAP1 in cystic and budding WT organoids grown in WT-cM or T-cM for 24 hr, in WT organoids overexpressing Thbs1 (lenti-Thbs1) or tumoroids, as indicated. (**P**) Quantification of the percentage of nYAP^HIGH^ cells/organoid in WT organoids cultured with T-cM and control IgG1 or anti-THBS1 (A6.1) antibodies for 24 hr. Scale bars correspond to 100 µm in (**G, I–N**). Graphs indicate average values ± SD. Statistical analysis was performed with two-tailed unpaired Welch’s *t*-tests. For the lenti-Thbs1 sample, a Welch’s corrected *t*-test was applied to compare the percentage of nYAP^HIGH^ cells/organoid between Thbs1-expressing organoids and WT organoids infected with an empty lentivirus. Figure 4—source data 1.Source data related to Figure 4H. Figure 4—source data 2.Source data related to Figure 4O. Figure 4—source data 3.Source data related to Figure 4P.

In order to decipher the molecular responses of WT epithelial cells to T-cM, we obtained the gene expression profiles of WT organoids exposed to either T-cM or WT-cM, along with the transcriptional signature of the tumoroids from which the corresponding T-cM was derived. Interestingly, we found that T-cM induced transcriptional responses enriched for genes upregulated in cancer, including colorectal adenoma (Figure 4—figure supplement 1A, red), suggesting that, alongside the typical tumour-like cystic morphology, WT cells also acquire signatures characteristics of tumour cells when exposed to T-cM. Furthermore, this analysis revealed that organoids grown in the presence of T-cM showed enrichment of genes of the Yes-associated protein (YAP)/Hippo pathway (Figure 4—figure supplement 1A, green). Given the intricate relationship between Wnt signalling and YAP in the intestine, suggesting that tumour formation requires additional signals other than Wnt, that induce YAP nuclear translocation (Cai et al., 2015; Azzolin et al., 2014; Gregorieff et al., 2015; Taniguchi et al., 2015; Taniguchi et al., 2017; Guillermin et al., 2021), we assessed the involvement of the YAP pathway in T-cM-mediated phenotypes. First, we compared our RNA-sequencing results to a YAP activation signature from intestinal organoids (Gregorieff et al., 2015) using Gene Set Enrichment Analysis (GSEA) and found a strong correlation with both WT organoids (Figure 4A) and tumoroids (Figure 4D). Consistent with recent studies describing YAP activation as an integral part of the regenerative and foetal programmes of the normal intestinal epithelium (Yui et al., 2018), we found a robust association with the reported physiological ‘foetal human colitis’ intestinal signature (Yui et al., 2018; Figure 4B and E), suggesting a link between the morphological change we characterised and reactivation of regenerative/foetal programmes occurring during tumorigenesis. These findings indicate that WT organoids in the presence of tumour-secreted factors, including THBS1, switch their transcriptional programme from a Wnt-dependent homeostatic to a Wnt-independent, YAP-dependent regenerative/foetal-like response, repressing differentiation genes (Figures 1I, 4C and F) without significantly affecting Wnt signalling (Figure 1F, Figure 4—figure supplement 1B).

**Figure 4—figure supplement 1.**
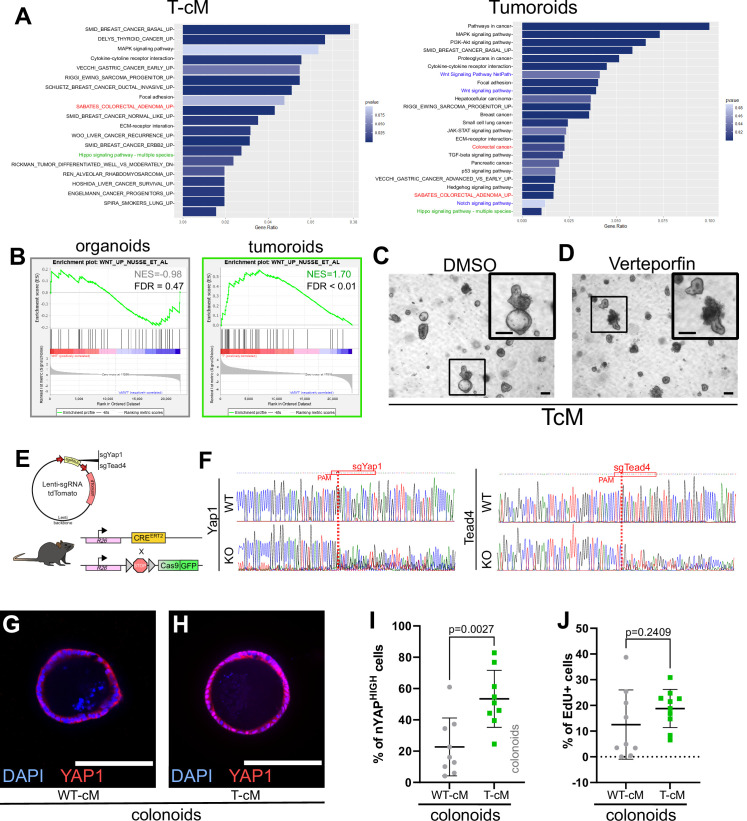
Related to Figure 4. (**A**) Pathway analysis of selected KEGG and GSEA-MSigDB terms enriched in the signatures of organoids grown in tumour conditioned medium (T-cM) (left panel) or tumoroids (right panel). (**B**) Gene Set Enrichment Analysis (GSEA) comparing differentially expressed genes in wildtype (WT) organoids exposed to T-cM or in tumoroids with the WNT signature (Nusse Lab). Green NES: positive correlation; grey NES: non-significant correlation. (**C, D**) Representative bright-field images of WT organoids grown in T-cM in the presence of DMSO (**C**) or the YAP inhibitor verteporfin (**D**). (**E**) Map of the lenti-sgRNA-tdTomato lentiviral vector used for knockout experiments and schematic diagram of the RosaCRE^ERT2^;Cas9-GFP mouse line used to derive WT organoids. (**F**) Analysis of CRISPR edits from Sanger sequencing of DNA at the expected cut site for Cas9 in mouse embryonic fibroblasts infected with no sgRNA (WT) or sgRNAs targeting Yap1 (KO in **F, **left) and Tead4 (KO in **F, **right). Red boxes indicate the sgRNA sequences with the PAM sequence underlined in red; red vertical dashed lines indicate the expected cut site. (**G, H**) Representative immunofluorescence against YAP1 (in red) of colonoids exposed to WT-cM (**G**) or T-cM (**H**) for 24 hr. DAPI stains DNA in blue. (**I, J**) Quantification of the percentage of YAP^HIGH^ cells/organoid based on the ratio of nuclear vs. cytoplasmic YAP1 (**I**) or of EdU+ cells/organoid (**J**) in WT colonoids grown in WT-cM or T-cM. Scale bar = 100 µm. Statistical analysis was performed with two-tailed unpaired Welch’s *t*-tests. Figure 4—figure supplement 1—source data 1.Source data related to Figure 4—figure supplement 1I. Figure 4—figure supplement 1—source data 2.Source data related to Figure 4—figure supplement 1J.

To assess the functional significance of YAP pathway activation, we pharmacologically blocked it using verteporfin, an inhibitor of the YAP-TEAD interaction (Liu-Chittenden et al., 2012), and observed a complete loss of cystic organoids with no discernible effects on their growth (Figure 4—figure supplement 1C and D). We further corroborated these results through the genetic deletion by CRISPR/Cas9 of Yap1 and one of its cellular effectors, the transcription factor Tead4 (Guillermin et al., 2021), found upregulated upon exposure to T-cM (Figure 4—figure supplement 1E and F). Yap1 or Tead4 knockout in WT organoids (Figure 4G) caused a considerable decrease in the proportion of cystic organoids induced by T-cM, suggesting that the YAP/Hippo pathway mediates the morphological change (Figure 4H). We further found that WT organoids, upon T-cM exposure, displayed a higher number of cells with nuclear YAP1, a readout of YAP pathway activation and a typical characteristic of tumoroids (Figure 4I–K and O). Notably, T-cM induces nuclear YAP accumulation in both cystic and budding organoids (Figure 4J, K and O), suggesting that YAP activation is necessary but not sufficient to induce the switch to the cystic phenotype.

Importantly, neutralisation of THBS1 with three blocking antibodies was sufficient to rescue both the cystic phenotype and YAP nuclear accumulation since the proportion of cells presenting nuclear YAP dropped to control levels (Figure 4L and P). Corroborating the key role of THBS1 in YAP activation, we also found that lentiviral overexpression of THBS1 (Lenti-Thbs1) was sufficient to trigger both cystic shapes and YAP nuclear translocation (Figure 4M and O). Furthermore, we assayed the effect of T-cM onto organoids derived from mouse colon (colonoids) and confirmed that T-cM also induced YAP activation and promoted proliferation in colonoids (Figure 4—figure supplement 1G–J), like in small intestinal organoids, consolidating the relevance of our findings to colon cancer.

### Thbs1-expressing and YAP-activated tumour cells are mutually exclusive in mouse tumours

**Figure 5.**
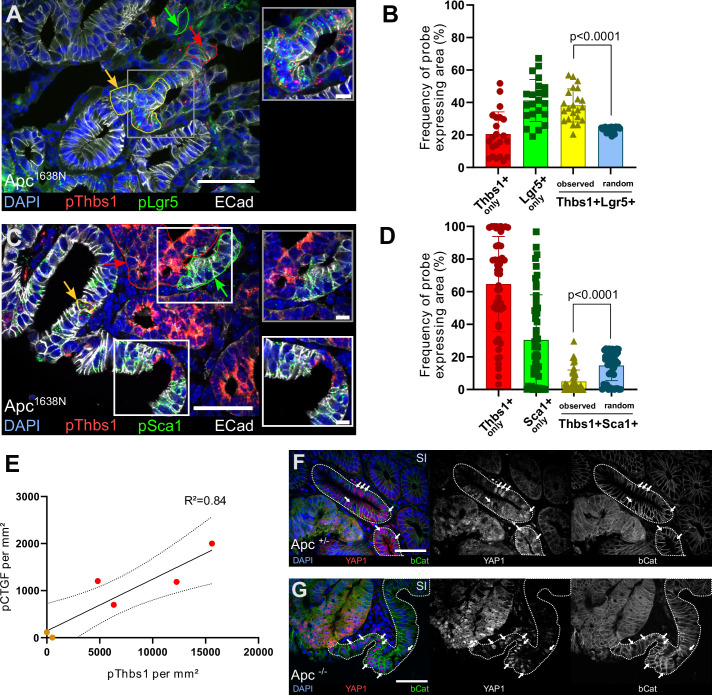
Thbs1 is expressed by Lgr5+ cancer stem cells in vivo and induces YAP activation in neighbouring epithelial cells. (**A, C**) Representative section of *Apc* mutant intestinal tumours analysed by single-molecule fluorescence in situ hybridisation (smFISH) for Thbs1 (pThbs1, red dots) and Lgr5 (pLgr5, green dots in **A**) or the YAP target Sca1 (pSca1, green dots in **C**). Examples of segmented and processed region of interest (ROI) that were automatically counted as co-localisation (Thbs1+/Lgr5+ in **A **or Thbs1+/Sca1+ cells in **C **outlined in yellow and indicated by yellow arrows) or single-probe expression (outlined in red or green and indicated by arrows of the corresponding colour) are shown. E-cadherin demarcates epithelial cells in white and DAPI labels nuclei in blue in (**A**) and (**C**). (**B, D**) Quantification of the frequency of tumour regions expressing exclusively one probe or co-expressing two probes (yellow): Thbs1 only in red or Lgr5 only in green (**B**); Thbs1 only in red or Sca1 only in green (**D**). The observed frequencies of co-localisation (yellow in **B**) or mutual exclusion (yellow in **D**) are statistically significant compared to the calculated probability of random co-expression (blue columns) (n = 22 sections from two tumours in **B **and n = 51 sections from five tumours in **D**). (**E**) Correlation of the number of RNA molecules (dots/mm²) detected by single-molecule RNA fluorescence in situ hybridisation (smRNA FISH) for the YAP target CTGF and Thbs1 in mouse intestinal tumours. Red dots indicate large tumours (≥ 8 mm), orange dots small tumours (<8 mm). Dashed lines indicate 95% confidence intervals. (**F, G**) Representative sections of tumours derived from *Villin*^CreERT2^;*Apc*^flox/+^ (*Apc*^+/-^ in **F**) or *Villin*^CreERT2^;*Apc*^flox/flox^ (*Apc*^-/-^ in **G**) immunostained for YAP1 (in red) and β-catenin (in green). Wildtype (WT) glands displaying membrane-bound β-catenin, adjacent to mutant areas presenting diffuse cytoplasmic/nuclear β-catenin expression are demarcated by dashed lines. White arrows indicate examples of cells showing high levels of nuclear YAP. Scale bars = 50 µm and 10 µm in insets. Statistical analysis was performed with Wilcoxon test in (**B–D**) and linear regression test with 95% confidence in (**E**). Figure 5—source data 1.Source data related to Figure 5B. Figure 5—source data 2.Source data related to Figure 5D. Figure 5—source data 3.Source data related to Figure 5E.

To further substantiate the in vivo relevance of our results, we induced acute *Apc* loss in *Villin*^CreERT2^;*Apc*^flox/flox^ mice for a short time (4 days) and found that Thbs1 was ectopically expressed by *Apc* mutant intestinal epithelial cells, indicating that Thbs1 expression is induced by Wnt activation (Figure 5—figure supplement 1A–C). To demonstrate that YAP nuclear accumulation is induced in WT epithelial cells neighbouring mutant tumour cells, we induced mosaic *Apc* loss in both *Villin*^CreERT2^;*Apc*^flox/+^ and *Villin*^CreERT2^;*Apc*^flox/flox^ mice, allowing us to study non-recombined WT epithelial cells adjacent to or within *Apc* mutant tumours. These experiments showed that in both *Apc* heterozygotes (Figure 5F) and homozygotes (Figure 5G) mice, the majority of the cells presenting nuclear YAP do not coincide with *Apc* mutant cells (displaying high levels of cytoplasmic and nuclear β-catenin, indicative of Wnt activation), but they are always in close proximity to mutant cells.

**Figure 5—figure supplement 1.**
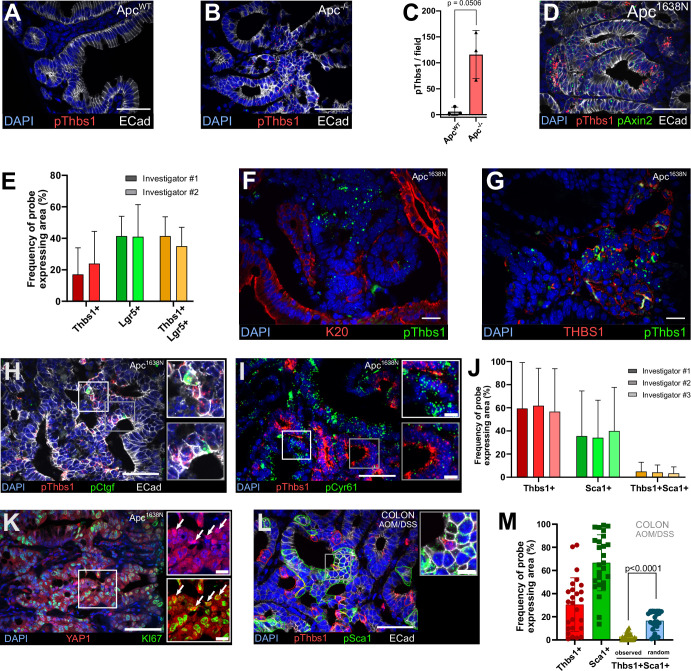
Related to Figure 5. (**A, B**) Representative sections of single-molecule RNA fluorescence in situ hybridisation (smRNA FISH) targeting Thbs1 (pThbs1, red dots) in the small intestine of wildtype (WT) mice (*Apc*^WT^ in **A**) or *Villin*^CreERT2^;*Apc*^flox/flox^ mice (*Apc*^-/-^ in **B**). (**C**) Quantification of the number of Thbs1 dots per field in *Apc*^WT^ or *Apc*^-/-^ small intestine. (**D**) Representative sections of smRNA FISH targeting Thbs1 (pThbs1, red dots) and the Wnt target Axin2 (pAxin2, green dots) in mouse intestinal tumours (*Apc*^1638N^). (**E**) Quantification of the percentage of probe-expressing area reflecting cells expressing Thbs1 only (red), Lgr5 only (green), or co-expressing both (yellow) annotated by three independent and double-blinded investigators, corresponding to Figure 5A and B. (**F, G**) Representative sections of mouse intestinal tumours analysed by smRNA FISH for Thbs1 (pThbs1, green dots) and immunostained with an antibody against the epithelial marker Keratin 20 (K20 in red in **F**) or with anti-THBS1 antibody (in red in **G**). (**H, I**) Representative sections of *Apc* mutant intestinal tumours analysed by smRNA FISH for the YAP target genes Ctgf (pCtgf, green dots in **H**) or Cyr61 (pCyr61, green dots in **I**) and Thbs1 (pThbs1, red dots). (**J**) Quantification of the percentage of probe-expressing area reflecting cells expressing Thbs1 only (red), Sca1 only (green), or co-expressing both (yellow) annotated by three independent and double-blinded investigators, corresponding to Figure 5C and D. (**K**) Representative sections of *Apc* mutant intestinal tumours immunostained for YAP1 (in red) and KI67 (in green). White arrows in insets show nuclear YAP1^HIGH^/Ki67+ cells (n = 6 sections from three tumours). (**L**) Representative sections of chemically induced colon tumours analysed by smRNA FISH for expression of Thbs1 (pThbs1, red dots) and the YAP target gene Sca1 (pSca1, green dots); regions presenting co-localisation of Thbs1 and Sca1 are outlined in yellow. (**M**) Quantification of the percentage of probe-expressing area reflecting cells expressing Thbs1 only (red), Sca1 only (green), or both (yellow). The observed mutual exclusion of red and green regions is statistically significant compared to the calculated probability of random co-expression (blue column) (n = 30 sections from five tumours). E-cadherin antibody in white demarcates epithelial cells in (**A, B, D, H, L**). DAPI labels nuclei in blue. Scale bar = 50 µm (10 µm in the insets). Statistical analysis was performed with two-tailed unpaired Welch’s *t*-tests in (**C**) (p=0.0506) and Wilcoxon test in (**M**). Figure 5—figure supplement 1—source data 1.Source data related to Figure 5—figure supplement 1E. Figure 5—figure supplement 1—source data 2.Source data related to Figure 5—figure supplement 1J. Figure 5—figure supplement 1—source data 3.Source data related to Figure 5—figure supplement 1M.

Consistent with these findings, we found that Thbs1+ cells in mouse tumours largely coincide with the cells expressing the widely accepted Wnt target genes Axin2 (Figure 5—figure supplement 1D) and Lgr5 (Barker et al., 2009; Figure 5A and B, Figure 5—figure supplement 1E), while they do not express the differentiation marker Keratin 20 (Figure 5—figure supplement 1F). The RNA probe recognising Thbs1 co-localises with the THBS1 protein visualised by antibody staining (Figure 5—figure supplement 1G). Furthermore, single-molecule RNA fluorescence in situ hybridisation (smRNA FISH) showed a clear and highly significant mutual exclusion between Thbs1-expressing cells and cells showing YAP activation, as assessed by expression of the YAP targets Ctgf (Figure 5—figure supplement 1H), Cyr61 (Figure 5—figure supplement 1I), Sca1 (Figure 5C and D, Figure 5—figure supplement 1J), and indicating the presence of two distinct tumour cell populations: one Thbs1+/Sca1- (64.61% ± 29.14%) and one Thbs1-/Sca1+ (30.40% ± 27.72%) (Figure 5D). Of relevance to colon cancer, these results are confirmed both in small intestinal adenomas (*Apc*^1638N^ in Figure 5 and Figure 5—figure supplement 1A–K) and chemically induced colon tumours (Figure 5—figure supplement 1L and M). At the whole-tumour scale, Thbs1 and CTGF expression are highly correlated (Figure 5E and R² = 0.84).

### The THBS1-YAP axis is conserved in early stages of human colorectal cancer

**Figure 6.**
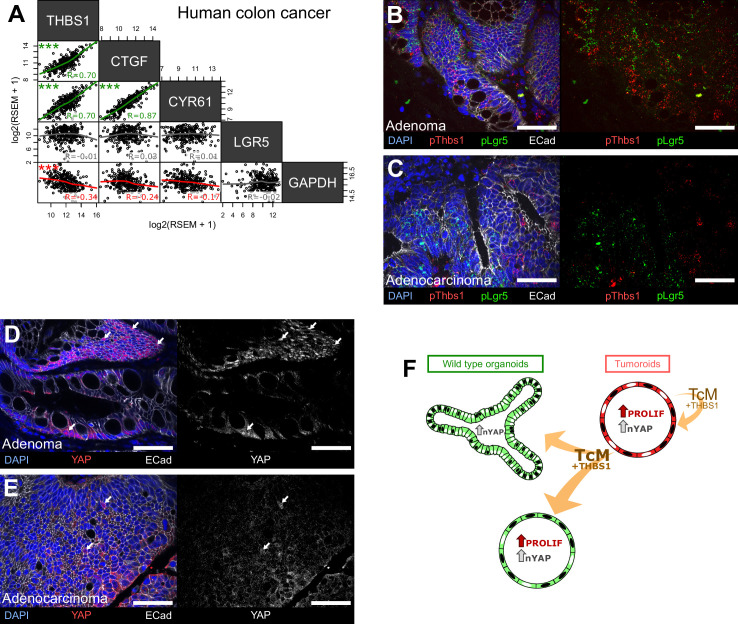
The THBS1-YAP pathway operates in human low-grade adenomas. (**A**) Correlation matrix between the expression levels of THBS1 and the YAP targets CTGF, CYR61, and LGR5 in human colon tumours from the TCGA colon cancer bulk datasets. R indicates Spearman’s coefficient. (**B–E**) Representative sections of low-grade human adenomas (**B, D**) or advanced human carcinomas (**C, E**) processed by single-molecule RNA fluorescence in situ hybridisation (smRNA FISH) for Thbs1 (pThbs1, red dots) and Lgr5 (pLgr5, green dots in **B, C**) or immunostained with anti-YAP1 antibodies (**D, E**). White arrows highlight tumour cells presenting high nuclear YAP in (**D, E**). n = 5 human low-grade adenomas in (**B, D**) and n = 5 advanced human adenocarcinomas in (**C, E**). (**F**) Graphical summary of paracrine interactions between wildtype (WT) organoids and tumoroids along the THBS1-YAP axis. Mutant tumoroids ‘corrupt’ genetically WT organoids by secreting THBS-1 (orange arrows). This results in YAP1 nuclear translocation (black nuclei in organoids or tumoroids) and ectopic proliferation as well as cystic morphology in a subset of organoids.

The main components of the signalling axis we have uncovered, *Thbs1*, *Ctgf,* and *Cyr61*, but not the Wnt target gene *Lgr5*, are highly correlated in bulk transcriptomics of human colorectal samples (Figure 6A). To establish if the mechanism mediating paracrine cellular communication that we uncovered is conserved in human colon cancer, we then analysed a cohort of 10 human colon tumours (five low-grade adenomas and five invasive carcinomas) for their expression of THBS1, LGR5, and YAP (Figure 6B–E). Supporting our results in mouse adenomas, the analysis of human tumours at different stages revealed that Thbs1 is highly expressed in Lgr5+ cells only in early-stage adenomas (Figure 6B) but not in advanced carcinomas (Figure 6C). Extensive co-expression of Thbs1 and Lgr5 in adenomas is accompanied by the presence of large tumour regions rich in cells presenting nuclear YAP (Figure 6D), which were not visible in invasive adenocarcinomas (Figure 6E). This intriguing observation can explain why no significant correlation between Thbs1 and Lgr5 expression was found in our in silico analysis of human advanced colon cancer (Figure 6A). These results, combined with our findings in organoids and transgenic mice, suggest a key role of the Thbs1-YAP axis in tumour initiation.

## Discussion

Our results implicate that both in intestinal tumours derived from spontaneous *Apc* loss and chemically induced colon tumours, cancer cells can directly recruit surrounding epithelial cells through Wnt-driven expression and secretion of the glycoprotein THBS1, which results in aberrant activation of a regenerative/foetal transcriptional programme mediated by the YAP pathway (Figure 6F), a driver of intestinal regeneration and tumorigenesis (Gregorieff et al., 2015). THBS1 is overexpressed in a large number of solid tumours, but its role in cancer is controversial. Constitutive deletion of Thbs1 in *Apc*^Min/+^ mice led to an increase in the number and aggressiveness of tumours, which was interpreted as a consequence of its anti-angiogenic role (Gutierrez et al., 2003). In human patients, consistent with our results supporting a role for THBS1 in tumour initiation but not progression (Figure 6), low expression of THBS1 has been found to correlate with more advanced grades of liver metastases derived from colorectal cancer after surgery, presence of lymph node metastases, and poor prognosis (Teraoku et al., 2016). However, THBS1 has been reported to promote the attachment of cells to the extracellular matrix, favouring cancer cell migration and invasion (Sid et al., 2008; Tuszynski et al., 1987). Indeed, a study using a model for inflammation-induced colon carcinogenesis (azoxymethane [AOM]/dextran sulphate sodium [DSS]) in Thbs1^-/-^ mice showed a fivefold reduction in tumour burden, suggesting a role for THBS1 in tumour progression (Lopez-Dee et al., 2015). These contradictory results are most likely due to the multifaceted effects of THBS1, depending on which cells secrete it and which cells respond. Of interest, a recent study proposed that THBS1 induces focal adhesions (FAs) and nuclear YAP translocation through interaction with αvβ1 integrins in the aorta (Yamashiro et al., 2019). Moreover, FAs have been shown to directly drive YAP1 nuclear translocation in the foetal intestine and upon inflammation in adult colon (Yui et al., 2018).

Here, we found that cancer cell-derived THBS1 can ‘corrupt’ WT epithelial cells in organoids, independently of stroma-derived effects, allowing us to address the specific role of THBS1 on epithelial cells. Surprisingly, we found that three neutralising antibodies targeting different epitopes of THBS1 were all able to block its effect on WT organoids. These results may indicate that THBS1 neutralisation is not due to block of a specific ligand-receptor interaction but rather to the steric interference with the trimerisation of the large soluble THBS1 isoform (450 kDa) that may no longer be free to diffuse through the Matrigel. Alternatively, it is possible that several THBS1 domains are involved in YAP activation, consistent with reports indicating that different THBS1 domains interact with integrins (Resovi et al., 2014).

Our results, corroborated by consistent observations in mouse and human intestinal tumours, uncovered a novel function for the secreted multidomain glycoprotein THBS1 in affecting the behaviour of normal epithelial cells surrounding a nascent tumour. The coexistence of WT and mutant cells within emerging tumours has been the subject of recent interest: consistent with our results, paracrine communication between epithelial cells has been found to induce YAP pathway activation (Flanagan et al., 2021; Yum et al., 2021; van Neerven et al., 2021; Krotenberg Garcia et al., 2021). However, it is still debated whether YAP has a tumour suppressor (Barry et al., 2013; Cheung et al., 2020) or oncogenic role (Zanconato et al., 2016). Indeed, while the recent studies cited above suggest that cancer cells actively eliminate WT cells by cell competition, facilitating tumour expansion, our results indicate that the recruitment of WT cells by cancer cells happens at the very early steps of tumour formation and may be required for the cancer cells to seed within a hyperplastic epithelium. However, consistent with the recent literature and our analysis of human tumours, at later stages of tumorigenesis, the fitter mutant cells outcompete WT cells, as shown by the decrease in cells expressing both THBS1 and nuclear YAP in advanced human adenocarcinomas (Figure 6C and E). We thus believe that the observed differences in cell behaviour could depend on the kinetics of the effects of tumour-secreted factors on WT cells, distinguishing very early responses (within 24–48 hr) analysed in this study and later outcomes (van Neerven et al., 2021). Also, only some WT cells, responding to tumour-secreted factors by activating YAP, may be able to survive within tumours, while the majority of WT cells would be outcompeted, as proposed by Krotenberg Garcia et al., 2021.

Based on the results we report here, we propose a model for tumour initiation where mutant cells can ‘corrupt’ surrounding WT epithelial cells by secreting THBS1, leading to YAP activation in the receiving cells. Through the paracrine mechanism we unravelled, causing reactivation of a regenerative foetal-like transcriptional programme, WT epithelial cells initially thrive in nascent tumours. In such a scenario, normal cells within tumours, which would escape specific therapies targeting mutant cells, could be identified by their hallmark of YAP activation, providing a novel diagnostic tool. Of relevance, THBS1 neutralisation showed a tumour-specific toxicity; we thus propose that THBS1 may represent a therapeutic target for colon cancer, potentially applicable to other epithelial tumours.

## Materials and methods

### Statistics and reproducibility

Experiments were performed in biological and technical replicates as stated. For each experiment, we have used at least n = 3 organoid lines originating from n = 3 different mice, and experiments with at least n = 3 replicates were used to calculate the statistical value of each analysis. All graphs show mean ± SD. Statistical analysis was performed with two-tailed unpaired Welch’s *t*-tests, unless otherwise stated.

### Transgenic mouse models

All mouse lines used have been previously described. Intestinal tumours were generated in *Apc*^1638N^ mutant mice (Fodde et al., 1994), crossed to the R26^mTmG^ line (Muzumdar et al., 2007), or in *Villin*^CreERT2^ (el Marjou et al., 2004) crossed to *Apc* Delta14 (Colnot et al., 2004) mice, and Lgr5-GFP (Barker et al., 2007) mice were kindly provided by H. Clevers. GFP-expressing wildtype organoids were generated from the LifeAct-GFP mouse line (Riedl et al., 2008). Organoids used for KO experiments were obtained by crossing R26-LSL-Cas9-GFP (Platt et al., 2014) and R26CreERT2 mouse lines (Ventura et al., 2007). All mice used were of mixed genetic background.

### Chemically induced colon tumour model

#### AOM/DSS colon carcinogenesis experimental protocol

To induce colon tumours, we followed the protocol from Tanaka et al., 2003: Notch1-Cre^ERT2^/R26^mTmG^ mice of 5–7 months of age received a single intraperitoneal injection of AOM (Sigma #A5486) followed by DSS (MP Biomedicals #160110) administration (3% in drinking water) 1 day after the AOM injection for five consecutive days. General health status and mouse body weight were monitored daily during and after treatment. To verify the presence of colon tumours, two mice were checked 1 month after the first cycle of DSS treatment, but no tumours were detected (only signs of inflammation). We administered another cycle of DSS (3% in drinking water) for 3 days, and tumour formation was monitored by colonoscopy using a Karl Storz endoscopic system.

### Human tumours

Five low-grade adenomas and five invasive adenocarcinomas were obtained from the Centre of Biological Resources of Institut Curie and examined by the service of pathology.

### Organoids cultures

#### Wildtype organoids

Wildtype organoids were cultured and passaged as previously described (Sato et al., 2009) and derived from the small intestine or colon of 2–5-month-old mice. The Matrigel crypts mix was plated as 50 µl drops in 24-well plates or 35 µl drops in eight-well Ibidi imaging chambers (Ibidi 80827) for whole-mount staining. After polymerisation, the Matrigel drop was covered with wildtype organoid medium containing EGF, Noggin, R-spondin1 (ENR) for small intestinal organoids or EGF, Noggin, R-spondin1, CHIR99021, Y27632, Wnt3a (ENRCYW) for colonoids. Reagents, media, and buffers are listed in the Key resources table. Factors, inhibitors, and neutralising antibodies that were added to the medium are also indicated in the Key resources table.

#### Tumoroids

*Apc*^1638N^ heterozygous mice of more than 6 months of age were dissected and intestinal tumours were harvested using forceps and micro-dissection scissors to reduce contamination with adjacent healthy tissue. Periampullary tumours were excluded from the study to avoid contamination with stomach cells. To remove the remaining healthy tissue surrounding the extracted tumour, tumours were incubated in 2 mM EDTA in PBS (pH = 8.0) for 30 min at 4°C. Tumours were then briefly vortexed to detach the remaining normal tissue, leaving clean spheres. In order to dissociate tumour cells, the tumour was chopped into 1–3 mm fragments using a razor blade and digested in 66% TrypLE (Thermo Fisher 12605010) diluted in PBS, for 10 min at 37°C under continuous agitation at 180 rpm. The supernatant containing the dissociated cells was harvested, and fresh 66% TrypLE was added to the remaining fragments for another 10 min. The supernatant was strained using a 70 µm cell strainer. Cells were then centrifuged at 400 × *g* for 5 min at 4°C, suspended in DMEM-F12 (2% Penicillin-Streptomycin) and plated in 50% Matrigel drops as described for wildtype organoid cultures. After polymerisation, 300 µl of EN medium containing EGF and Noggin (EN) was added. The medium was replaced every 1–2 weeks. Tumoroids were passaged every 1 (for line expansion) or 3–4 weeks (for medium conditioning). Reagents and medium composition are listed in the Key resources table.

### Co-culture assay

Wildtype and tumour organoid cultures were started at least 2 weeks before co-culture in order to use stable and exponentially growing cultures. These passages guaranteed morphologically homogeneous organoids. After passage of wildtype and tumour organoids, the fragments were mixed at approximately 3:1 wildtype organoid to tumoroids ratio. Mixed fragments were plated as described above. After Matrigel polymerisation at 37°C, ENR medium (300 µl/well) was added. Reagents and medium composition are listed in the Key resources table.

### Conditioned medium assay

WT organoids were passaged as previously described. After Matrigel polymerisation, 150 µl of ENR 2× concentrated and 150 µl of cM were added. Analyses were performed between 24 and 48 hr after plating, unless otherwise specified.

### Tumoroid conditioned medium preparation

Established tumoroid cultures (more than two passages) were grown for 1 week. After 1 week of expansion, fresh medium was added and conditioned for 1–2 weeks depending on the organoid density. Immediately after harvesting, the cM was centrifuged at 400 × *g* for 5 min at 4°C to remove big debris and cell contamination. The supernatant was recovered and centrifuged again at 2000 × *g* for 20 min at 4°C and then ultracentrifuged at 200,000 × *g* for 1.5 hr at 4°C in order to remove extracellular vesicles. The supernatant was snap-frozen in liquid nitrogen and stored at –80°C.

### WT conditioned medium preparation

WT intestinal organoid cultures were passaged and grown for 3 days in order to obtain high-density cultures. Then, fresh ENR medium was conditioned for 1 week and prepared as described above.

### Organoid freezing

Matrigel drops containing organoids in exponential growth were collected in PBS and centrifuged twice at 400 × *g* for 5 min at 4°C to remove Matrigel and debris. The organoids were suspended at a high density in Cryostor10 freezing medium (six wells of organoids per 1 ml of cryogenic medium). Organoids were incubated for 10 min in Cryostor10 before being frozen.

### Organoid thawing

Organoids were quickly thawed at 37°C and suspended in 5 ml of FBS prior to centrifugation at 400 × *g* for 5 min at 4°C. The organoids were suspended in DMEM-F12 with 2% Penicillin/Streptomycin and mixed with Matrigel at a 1:1 ratio as described above. After polymerisation at 37°C, ENR or EN medium was added. Due to the FBS impact on organoid morphology, the thawed organoids were passaged at least once and carefully checked for their morphology prior to use. Reagent and medium composition are listed in the Key resources table.

### Organoid infection

Wildtype or tumour organoids were put in culture at least 1 week before viral transduction. To plate 8 wells of infected organoids, 12 wells of exponentially growing organoids were harvested in cold Cell Recovery Solution and incubated on ice for 15 min to dissolve the Matrigel. Organoids were then centrifuged at 400 × *g* for 5 min at 4°C. For cell dissociation, the pellet was suspended in 2 ml of AccuMax (Sigma A7089) containing CHIR99021, Y27632 (CY), and incubated at 37°C for 8 min. Digestion was then stopped by adding 2 ml of DMEM-F12 containing B27 and CY. Organoids were further mechanically dissociated by pipetting up and down 40–50 times. The cell suspension was then centrifuged at 400 × *g* for 5 min at 4°C. Organoids were carefully suspended in 300 µl of 66× concentrated virus (see below) containing CY and TransDux reagents prior to addition of 300 µl of cold-liquid Matrigel and plated as previously described. After polymerisation at 37°C, 300 µl/well of ENR-CY medium was added. ENR-CY was replaced by ENR 2 days later. This is essential to avoid a morphological change to cysts due to exposure to CHIR99021. After 4 days, organoids were passaged and cultured as described above. Reagents and medium composition are listed in the Key resources table.

### Lentiviruses

#### Plasmids

Lenti-7TG was a gift from Roel Nusse (Addgene plasmid# 24314; http://n2t.net/addgene:24314; RRID:Addgene_24314). The Lenti-sgRNA-mTomato (LRT) construct was obtained by replacement of the GFP sequence with tandem Tomato in the Lenti-sgRNA-GFP (LRG), a gift from Christopher Vakoc (Addgene plasmid# 65656; http://n2t.net/addgene:65656; RRID:Addgene_65656). LentiCRISPRv2GFP was a gift from David Feldser (Addgene plasmid# 82416; http://n2t.net/addgene:82416; RRID:Addgene_82416). LentiThbs1Tg is a lentiORF-expressing mouse Thbs1 (NM_011580) –myc-DKK (Origene# MR211744L3V). pMD2.G was a gift from Didier Trono (Addgene plasmid# 12259; http://n2t.net/addgene:12259; RRID:Addgene_12259). psPAX2 was a gift from Didier Trono (Addgene plasmid# 12260; http://n2t.net/addgene:12260; RRID:Addgene_12260).

#### sgRNA cloning

Both lentivirus backbones used for the knockout experiments harboured the GeCKO cloning adaptors (Shalem et al., 2014; Sanjana et al., 2014). The sgRNAs inserted in either vectors are listed in the Key resources table.

#### Virus production

Lentiviral particles were produced in HEK 293T cells. Day 0: 8 × 10^6^ cells were plated onto a T75 flask in 10 ml of complete medium. Day 1: cells were transfected using PEI/NaCl. For this, two solutions were prepared: mix A contained 625 µl NaCl 150 mM + 75 µl PEI and mix B contained 625 µl NaCl 150 mM + 6 µg of plasmid DNA at a molar ratio of 4:3:2 (lentiviral vector: psPAX2: pMD2.G). Mix A and B were incubated for 5 min at room temperature (RT) and then mixed and incubated for 15 min at RT before being added drop by drop on top of the cells. Medium was changed on day 2, and 10 ml of supernatant containing virus particles was collected on days 3 and 4. The supernatants were centrifuged for 5 min at 1500 rpm at 4°C in order to remove dead cells and debris. The virus particles were then concentrated to 300 µl using Amicon Ultra Centrifugal Filters (UFC910024; Sigma) by spinning at 1000 × *g* at 4°C for 1 hr.

#### sgRNA validation

The efficiency of sgRNAs was assessed in mouse embryonic fibroblasts (MEFs) infected with a lentivirus expressing the Cas9 enzyme along with blasticidin resistance (Addgene plasmid# 52962). 48 hr upon infection, infected MEFs were selected in 10 µg/ml blasticidin for 7 days. sgRNAs targeting Thbs1, Yap1, and Tead4 (sequences listed in the Key resources table) were cloned in the LRT lentiviral vector (expressing red Tomato fluorescent protein) and produced as described above. After transduction of the MEFs-Cas9 with LRT-Thbs1, Yap1, or Tead4, cells were FACS-sorted based on their red fluorescence and their genomic DNA extracted. For each sgRNA, the cut site region (± 200 bp) was PCR-amplified and sequenced using the same primers (listed in the Key resources table). Chromatograms were manually analysed using ApE (v2.0.61) to confirm the precise cut site, which induced mutations starting at –3 bp before the PAM sequence.

### Mass spectrometry

#### Sample preparation

Isotope-labelled cM requires pre-loading of isotopic amino acids in order to detect all proteins synthesised and secreted by cells (Ong et al., 2002). To label newly produced proteins, two essentials isotopically labelled amino acids (IAA), arginine and lysine, were added to the culture medium. WT organoids were labelled with [²H_4_]-lysine (Lys4) and [^13^C_6_]-arginine (Arg6) for 2 weeks (two passages), whilst tumoroids were labelled with [^13^C_6_^15^N_2_]-lysine (Lys8) and of [^13^C_6_^15^N_4_]-arginine (Arg10) for 2 weeks (two passages). Then, medium was conditioned as described above using SILAC-ENR (2 × 1 week) or SILAC-EN (1 × 2 weeks). After a functional assay confirming their transforming capacities, conditioned media were concentrated. Subsequently, 2 ml of tumoroids conditioned media or 4 ml of wildtype conditioned media were precipitated by cold acetone. Dried protein pellets were then recovered with 50 µl of 2× Laemmli buffer with SDS and β-mercapto-ethanol (0.1%), boiled at 95°C for 5 min and centrifuged 5 min at 14,000 × *g*.

#### MS sample processing

Gel-based samples were cut in eight bands and in-gel digested as described in standard protocols. Briefly, following the SDS-PAGE and washing of the excised gel slices, proteins were reduced by adding 10 mM dithiothreitol (Sigma-Aldrich) prior to alkylation with 55 mM iodoacetamide (Sigma-Aldrich). After washing and shrinking of the gel pieces with 100% acetonitrile, trypsin/LysC (Promega) was added and proteins were digested overnight in 25 mM ammonium bicarbonate at 30°C. Extracted peptides were dried in a vacuum concentrator at RT and re-dissolved in solvent A (2% MeCN, 0.3% TFA) before LC-MS/MS analysis.

#### LC-MS/MS analysis

Liquid chromatography (LC) was performed with an RSLCnano system (Ultimate 3000, Thermo Scientific) coupled online to an Orbitrap Fusion Tribrid mass spectrometer (MS, Thermo Scientific). Peptides were trapped on a C18 column (75 μm inner diameter × 2 cm; nanoViper Acclaim PepMap 100, Thermo Scientific) with buffer A′ (2/98 MeCN/H_2_O in 0.1% formic acid) at a flow rate of 2.5 µl/min over 4 min. Separation was performed on a 50 cm × 75 μm C18 column (nanoViper Acclaim PepMap RSLC, 2 μm, 100 Å, Thermo Scientific) regulated to a temperature of 55°C with a linear gradient of 5–30% buffer B (100% MeCN in 0.1% formic acid) at a flow rate of 300 nl/min during 100 min. Full-scan MS was acquired in the Orbitrap analyser with a resolution set to 120,000, a mass range of *m/z* 400–1500 and a 4 × 105 ion count target. Tandem MS was performed by isolation at 1.6 Th with the quadrupole, HCD fragmentation with normalised collision energy of 28, and rapid scan MS analysis in the ion trap. The MS2 ion count target was set to 2 × 104, and only those precursors with charge state from 2 to 7 were sampled for MS2 acquisition. The instrument was run at maximum speed mode with 3 s cycles.

#### Mass spectrometry data processing

Data were acquired using the Xcalibur software (v 3.0), and the resulting spectra were interrogated by SequestHT through Thermo Scientific Proteome Discoverer (v 2.1) with the *Mus musculus* Swissprot database (022017 containing 16,837 sequences and 244 common contaminants). The mass tolerances in MS and MS/MS were set to 10 ppm and 0.6 Da, respectively. We set carbamidomethyl cysteine, oxidation of methionine, N-terminal acetylation, heavy ^13^C_6_^15^N_2_-lysine (Lys8) and ^13^C_6_^15^N_4_-arginine (Arg10), and medium ^2^H_4_-lysine (Lys4) and ^13^C_6_-arginine (Arg6) as variable modifications. We set specificity of trypsin digestion and allowed two missed cleavage sites. The resulting files were further processed by using myProMS (v 3.5) (Poullet et al., 2007). The SequestHT target and decoy search results were validated at 1% false discovery rate (FDR) with Percolator. For SILAC-based protein quantification, peptide extracted ion chromatograms (XICs) were retrieved from Thermo Scientific Proteome Discoverer. Global MAD normalisation was applied on the total signal to correct the XICs for each biological replicate (n = 3). Protein ratios were computed as the geometrical mean of related peptides. To estimate ratio significance, a *t*-test was performed with the R package limma (Ritchie et al., 2015) and the FDR was controlled using the Benjamini–Hochberg procedure (Benjamini and Hochberg, 1995) with a threshold set to 0.05. Proteins with at least two peptides detected, a twofold enrichment, and an adjusted p-value<0.05 were retained as significant hits.

#### Pathway enrichment analysis

GO terms enrichment analysis used the proteins significantly enriched in sample comparisons (T-cM/WT-cM; two peptides, fold change > 2, adjusted p-value<0.05) and the unique proteins to T-cM. GO biological processes, cellular components, and molecular functions were analysed using the UniProt-GOA Mouse file (v. 20181203). Significant GO terms had a p<0.05.

### Immunofluorescence (IF)

#### Organoids staining

For whole-mount IF, organoids were grown on eight-well chamber slides (Ibidi 80827). For EdU staining, a 2 hr pulse of EdU (10 µM, Carbosynth Limited NE08701) preceded fixation. After fixation using 4% paraformaldehyde (Euromedex 15710) in PBS for 1 hr at RT, organoids were washed with PBS and permeabilised in PBS + 1% Triton X-100 (Euromedex 2000-C) for 1 hr at RT. Organoids were then incubated with 150 µl of diluted antibodies (listed in the Key resources table) in blocking buffer (PBS, 2% BSA, 5% FBS, 0.3% Triton X-100) overnight at RT. After three washes of 5 min in PBS, 150 µl of secondary antibodies were added together with DAPI diluted in PBS and incubated at RT for 5 hr. Organoids were then washed with PBS for three times for 5 min each and stored in a 1:1 ratio PBS and glycerol (Euromedex 15710) before imaging. For EdU staining, the EdU signal was revealed after the secondary antibody step using the EdU Click-it kit (Thermo Fisher Scientific C10340).

#### Human sections IF staining

Paraffin sections 3 µm of human samples were deparaffinised and rehydrated using the standard protocol of xylene/ethanol gradient. Antigens were unmasked by boiling the slides in a citrate-based solution (Eurobio-Abcys H-3300). Slides were then incubated in blocking buffer (PBS, 2% BSA, 5% FBS). Antibodies (listed in the Key resources table) were incubated overnight at 4°C. After three washes of 5 min each in PBS, secondary antibodies were incubated at RT for 2 hr alongside DAPI. Slides were mounted in Aqua-poly/mount (Tebu Bio 18606-5).

#### Mouse sections IF staining

Intestinal tissue samples were fixed overnight at RT with 10% formalin prior to the paraffin embedding. 4 µm FFPE sections were prepared for β-catenin/YAP co-staining. Briefly, the tissue sections were deparaffinised five times in xylene 5 min each, rehydrated five times in ethanol 100% 5 min each, then once in ethanol 70% for 10 min. A heat-mediating antigen retrieval was made using boiling sodium citrate tribasic dihydrate solution 10 mM, PH = 6 (Sigma S4641) for 20 min. The sections were then blocked and permeabilised with 5% donkey serum 0.01% Triton X-100 for 30 min at RT before being incubated with rabbit anti-YAP dilution 1/100 (Signaling Technology #14074) and mouse anti β-catenin (BD 610153) (dilution 1/200) overnight at 4°C. The next day, slides were washed three times in PBS Tween20 0.01% and then incubated for 1 hr at RT with matching secondary antibodies donkey anti-rabbit A594 (Jackson ImmunoResearch 711-546-152) and donkey anti-mouse A488 (Jackson ImmunoResearch 715-546-150) (dilution 1/500) with DAPI. Slides were mounted using Fluoromount Aqueous Mounting Medium (Sigma F4680).

#### Single-molecule RNA fluorescence in situ hybridisation

smRNA FISH was performed on mouse tissue cryosections or human paraffin-embedded tumour sections using RNAscope Multiplex Fluorescent Detection Kit v2 (ACD 323110) and pipeline following manufacturer’s recommendations. Thbs1 mRNA were labelled using RNAscope Probe- Mm-Thbs1-C3 (#457891-C3) or Hs-THBS1-C2 (#426581-C2), CTGF mRNA were labelled using RNAscope Probe- Mm-CTGF (#314541) and Lgr5 mRNA were labelled using RNAscope Probe- Mm-Lgr5 (#312171) or Hs-LGR5-C3 (#311021-C3). In order to subsequently perform immunostaining after the FISH, a protease III step not exceeding 20 min was included. Subsequent antibody staining was performed as described.

#### Epithelial masks generation

To quantify RNAscope results only in epithelial cells, epithelial masks were generated using E-cadherin immunostaining. After Ilastik training allowing segmentation of E-cadherin-stained membranes, masks were smoothed by closing function (iteration = 15) and holes filling. Generated masks were manually corrected for consistency.

#### smRNA FISH dots quantification

Raw images were segmented using Ilastik (v1.3.2) and training performed on negative controls (background), positive controls, and experimental slides (dots). Segmented masks were cleaned using Fiji through an opening function, 2 px Gaussian blur and Moments threshold. Generated masks were manually checked for consistency with raw data. Aggregates of dots were excluded using watershed function and individual dots (size = 2–250 circularity = 0.50–1.00) were analysed and counted using built-in Analyse Particle function. Epithelial dots were obtained by multiplication of dot masks by the corresponding epithelial masks previously generated.

#### Image acquisition

Images were obtained on an Inverted Wide Confocal Spinning Disk microscope (Leica) using ×40/1.3 OIL DIC H/N2 PL FLUOR or ×20/0.75 Multi Immersion DIC N2 objectives and Hamamtsu Orca Flash 4.0 camera. Images were captured using MetaMorph. Whole-plate acquisition was performed using a dissecting microscope and Cell Discoverer 7 (Leica). Images were captured with ZEN. For RNAscope experiments, images were obtained on a PLAN APO ×40/1.3 NA objective on an upright spinning disk (CSU-X1 scan-head from Yokogawa) microscope (Carl Zeiss, Roper Scientific, France), equipped with a CoolSnap HQ2 CCD camera (Photometrics). Images were captured using MetaMorph.

#### Nuclear/cytoplasmic quantification

Nuclear IF ratios were obtained using a custom-made ImageJ macro. This macro segmented the nuclei on the DAPI channel using Otsu Threshold, Watershed, and Particles analysis. For each segmented nucleus, a cytoplasmic halo of eight pixels was generated and excluded from the DAPI mask to avoid false cytoplasmic measurements in neighbouring nuclei. The mean intensities of the segmented nucleus and cytoplasmic regions of interest (nROI and cROI) were then measured in the IF channel (nIF and cIF). Cells were counted only if area ratio nucleus/cytoplasm > 0.5, avoiding bias of pixel sampling either due to miss-segmentation or to overcrowded regions. Results (nIF, cIF, and ratio) were computed in Microsoft Excel. A density curve of the nIF/cIF ratio was performed for each category of organoid in order to observe peaks trends of positive and negative nuclei for IF. Threshold was defined manually in the inter-peak region at 1.1 (YAP) and 1.25 (EdU). To exclude low or non-specific signal, a minimal mean intensity cut-off for cIF was established from the experimental images. Using the threshold and the cut-off, the percentage of nIF^HIGH^ cells per organoid was calculated.

### RNA-sequencing

#### Sample preparation

Organoids were harvested using Cell Recovery Solution as described above. After 15 min of Matrigel dissolution, organoids were pelleted at 500 × *g* for 5 min. Pellets were recovered in 1 ml of PBS in 1.5 ml tubes and pelleted again at same conditions. RNA extraction was performed using RNeasy Mini Kit (QIAGEN) following the manufacturer’s recommendations. Total RNA integrity (RINe) were subjected to quality control and quantification using an Agilent TapeStation instrument showing excellent integrity (RNA Integration Number, RIN = 10). NanoDrop spectrophotometer was used to assess purity based on absorbance ratios (260/280 and 260/230).

#### RNA-sequencing

RNA-sequencing libraries were prepared from 1 µg of total RNA using the Illumina TruSeq Stranded mRNA Library preparation kit that allows to prepare libraries for strand-specific mRNA-sequencing. A first step of polyA selection using magnetic beads was performed to address sequencing specifically on polyadenylated transcripts. After fragmentation, cDNA synthesis was performed followed by dA-tailing before ligation of the TruSeq indexed adapters (Unique Dual Indexing strategy). PCR amplification generated the cDNA library. After qPCR quantification, sequencing was carried out using 2 × 100 cycles (paired-end reads, 100 nucleotides) on an Illumina NovaSeq 6000 system (S1 flow cells) to get around 45 M paired-end reads per sample. FastQ files were generated from raw sequencing data using bcl2fastq where demultiplexing was performed according to indexes.

#### RNA-seq data processing

Sequencing reads were aligned on the Mouse Reference Genome (mm10) using the STAR mapper (v2.5.3a) (Dobin et al., 2013). Protein-coding genes from the Gencode annotation (vM13) have been used to generate the raw count table. Overall sequencing quality controls report a very high-sequencing quality, a high fraction of mapped reads, and a high enrichment in exonic reads.

#### Differential analysis

Expressed genes (TPM ≥ 1 in at least one sample) have then been selected for supervised analysis. The raw count table was normalised using the TMM method from the edgeR R package (v3.25.9) (Robinson et al., 2010), and the limma (Ritchie et al., 2015) voom (v3.39.19) functions were applied to detect genes with differential expression. In order to compare tumoroids versus wildtype samples, we designed a linear model as follows:

$$Y_{its}=\mu_{i}+T_{it}+E_{it}$$

where T is the type effect (T = {WT-cM, T-cM, Tumoroids}). We then restricted the dataset to WT-cM and T-cM samples, and applied the following model:

$$Y_{its}=\mu_{i}+T_{it}+S_{s}+E_{its}$$

where T is the type effect (T = {WT-cM, T-cM}) and S is the sample effect (S = {sample1, sample2, sample3}). All raw p-values were corrected for multiple testing using the Benjamini–Hochberg method (Benjamini and Hochberg, 1995). Genes with an adjusted p<0.05 and a log2 fold change >1 were called significant.

#### Pathway enrichment

We applied pathway enrichment analysis on upregulated genes (p-value<0.05 and logFC > 1) in T-cM and tumoroid samples compared to normal organoids (WT-cM) using KEGG, MSigDB curated gene sets, and MSigDB regulatory target gene sets. The enrichment analysis was performed using the R package clusterProfiler (v3.14.3) and msigdbr (v7.1.1).

Source code is available at https://gist.github.com/wenjie1991/d79fe428ac80c8f2e5d781a966df3978, (Jacquemin, 2022a copy archived at swh:1:rev:0887f17c2a830b5adfc42757a744a547a8e8fc54).

#### Gene Set Enrichment Analysis

GSEA v4.0.3 was used to generate and calculate the enrichment score. Transcriptional signatures used for the analysis were extracted from the literature (Gregorieff et al., 2015; Yui et al., 2018; Merlos-Suárez et al., 2011; Mourao et al., 2019) and Nusse Lab (https://web.stanford.edu/group/nusselab/cgi-bin/wnt/target_genes). GSEA were calculated by gene set and 1000 permutations in our RNASeq normalised reads count matrix.

#### Human colon cancer gene expression analysis

The gene expression data and clinical variables from the TCGA Colon Adenoma (COAD) cohort were downloaded from TSVdb (Sun et al., 2018) on 20 June 2020. Analyses were performed on Primary Solid Tumour gene expression data subset. Pairwise correlation analysis assayed THBS1, CTGF, CYR61, and WWC2 on 285 COAD tumour samples. The expression data were transformed by log2. Then, Spearman’s correlation was calculated and visualised by the PerformanceAnalytics (v2.0.4) R package.

Source code is available at https://gist.github.com/wenjie1991/6ff60b3edd5f61d0bd2ebe4f9404e46e, (Jacquemin, 2022b copy archived at swh:1:rev:5d5522e57aeb12b67347377e13e45fd3c1304836).

### Data and materials availability

The mass spectrometry proteomics data have been deposited to the ProteomeXchange Consortium via the PRIDE partner repository with the dataset identifier PXD020002 (Perez-Riverol et al., 2019). The RNA-sequencing data have been deposited in the Gene Expression Omnibus (GEO) repository under accession code GSE153160: whole-genome transcriptomic analysis of intestinal organoids and tumoroids. All other data supporting the conclusions of this study are provided in the main text or the supplementary materials.

## Data Availability

Sequencing data have been deposited in the Gene Expression Omnibus (GEO) repository under accession code GSE153160: Whole-genome transcriptomic analysis of intestinal organoids and tumoroids. Mass spectrometry proteomics data have been deposited to the ProteomeXchange Consortium via the PRIDE partner repository with the dataset identifier PXD020002. All data generated or analysed during this study are included in the manuscript and supporting files; a Source Data file has been provided for all Figures, including Figure supplements. The following datasets were generated: JacqueminG 2020Whole-genome transcriptomic analysis of intestinal organoids and tumoroidsGEOGSE153160 JacqueminG 2020Paracrine interactions between epithelial cells promote colon cancer growthProteomeXchangePXD020002

## Additional files

Transparent reporting form

## Appendix 1

**Appendix 1—key resources table:**

Reagent type (species) or resource	Designation	Source or reference	Identifiers	Additional information
Antibody	Anti-mouse Alexa Fluor 488 (donkey polyclonal)	Jackson ImmunoResearch	715-546-150	(1:500)
Antibody	Anti-mouse Alexa Fluor 488 (donkey polyclonal)	Jackson ImmunoResearch	711-546-152	(1:500)
Antibody	Anti-rabbit Alexa Fluor 488 (donkey polyclonal)	Thermo Fisher Scientific	A21206	(1:300)
Antibody	Anti-mouse Alexa Fluor 633 (donkey polyclonal)	Thermo Fisher Scientific	A21202	(1:300)
Antibody	Anti-rabbit Alexa Fluor 633 (goat polyclonal)	Thermo Fisher Scientific	A21071	(1:300)
Antibody	Anti-rabbit Cy3 (goat polyclonal)	Thermo Fisher Scientific	A10520	(1:300)
Antibody	Anti-rabbit Cy5 (goat polyclonal)	Thermo Fisher Scientific	A10523	(1:300)
Antibody	Anti-β-catenin (mouse monoclonal)	BD Transduction Laboratories	610153	(1:200)
Antibody	Anti-ANG (mouse monoclonal)	Abcam	ab10600	(2.5–25 μg/ml)
Antibody	Anti-E-cadherin (mouse monoclonal)	BD Transduction Laboratories	610182	(1:400)
Antibody	Anti-E-cadherin (rabbit monoclonal)	Cell Signaling Technology	3195	(1:300)
Antibody	Anti-FLAG (mouse monoclonal)	MilliporeSigma	F1804	(1 μg/ml)
Antibody	Anti-LGALS3 (mouse monoclonal)	Abcam	ab2785	(2.5–25 μg/ml)
Antibody	Anti-THBS1 (mouse monoclonal)	Novus Biologicals	2059SS	(1:100)
Antibody	Anti-THBS1 A4.1 (mouse monoclonal)	Thermo Fisher Scientific	MA5-13377	(5–20 μg/ml)
Antibody	Anti-THBS1 A6.1 (mouse monoclonal)	Novus Biologicals	NB100-2059	(5–20 μg/ml)
Antibody	Anti-THBS1 C6.7 (mouse monoclonal)	Thermo Fisher Scientific	MA5-13390	(5–20 μg/ml)
Antibody	IgG1 isotype control (MG1K) (mouse monoclonal)	Novus Biologicals	NBP1-96983	(5–20 μg/ml)
Antibody	Anti-cleaved Caspase3 (rabbit polyclonal)	Cell Signaling Technology	9661	(1:200)
Antibody	Anti-CP (rabbit polyclonal)	Abcam	ab48614	(2.5–25 μg/ml)
Antibody	Anti-CTGF (rabbit monoclonal)	R&D Systems	MAB91901-100	(1.25–12.5 μg/ml)
Antibody	Anti-HDGF (rabbit polyclonal)	Novus Biologicals	NBP1-71926	(0.5–5 μg/ml)
Antibody	Anti-Keratin 20 (rabbit monoclonal)	Cell Signaling Technology	13063	(1:200)
Antibody	Anti-Ki67 (rabbit polyclonal)	Abcam	ab15580	(1:200)
Antibody	Anti-LGALS3BP (rabbit polyclonal)	Abcam	ab217760	(2.5–25 μg/ml)
Antibody	Anti-YAP (rabbit monoclonal)	Cell Signaling Technology	14074	(1:100)
Antibody	Anti-TTR (sheep polyclonal)	Abcam	ab9015	(63–120 μg/ml)
Biological sample (*Homo sapiens*)	CRC adenocarcinoma	Centre of Biological Resources of Institut Curie		
Biological sample (*H. sapiens*)	Low-grade CRC adenoma	Centre of Biological Resources of Institut Curie		
Cell line (*H. sapiens*)	HEK293T	ATCC	12022001	
Cell line (*Mus musculus*)	MEF	PMID:34782763		MEFs derived from E13 wt embryo, a gift from Dr. Raphael Margueron
Chemical compound, drug	Aqua poly/mount	Tebu Bio	18606-5	Pure
Chemical compound, drug	[^13^C_6_]-arginine (Arg6)	MilliporeSigma	643440	1 µl/ml
Chemical compound, drug	[^13^C_6_^15^N_4_]-arginine (Arg10)	MilliporeSigma	608033	1 µl/ml
Chemical compound, drug	B27	Thermo Fisher Scientific	12587-010	1×
Chemical compound, drug	Cell Recovery Solution	Corning	354253	1×
Chemical compound, drug	CHIR99021	AMSBIO	1677-5	5 μM
Chemical compound, drug	Citrate-based solution	Vector Laboratories	H-3300	1×
Chemical compound, drug	Cryostor10	Stem Cell Technologies	07930	1×
Chemical compound, drug	DMEM	Thermo Fisher Scientific	31053028	1×
Chemical compound, drug	DMEM-F12	Thermo Fisher Scientific	11039-047	1×
Chemical compound, drug	DMEM F-12 FOR SILAC	Thermo Fisher Scientific	D1801047	1×
Chemical compound, drug	EDTA	MilliporeSigma	E6765	2 mM
Chemical compound, drug	EdU	Carbosynth Limited	NE08701	10 μM
Chemical compound, drug	FBS	Thermo Fisher Scientific	10500064	Pure
Chemical compound, drug	Fluoromount Aqueous Mounting Medium	MilliporeSigma	F4680	Pure
Chemical compound, drug	GlutaMAX	Thermo Fisher Scientific	35050038	1×
Chemical compound, drug	Glycerol	Euromedex	15710	50%
Chemical compound, drug	hiFBS	Thermo Fisher Scientific	10500064	10%
Chemical compound, drug	[²H_4_]-lysine (Lys4)	MilliporeSigma	616192	1 µl/ml
Chemical compound, drug	[^13^C_6_^15^N_2_]-lysine (Lys8)	MilliporeSigma	608041	1 µl/ml
Chemical compound, drug	[^13^C_6_^15^N_4_]-arginine (Arg10)	MilliporeSigma	608033	1 µl/ml
Chemical compound, drug	NaCl	MilliporeSigma	S9888	150 mM
Chemical compound, drug	Non-Essential Amino Acids	Thermo Fisher Scientific	11140035	1×
Chemical compound, drug	Paraformaldehyde	Euromedex	15710	4%
Chemical compound, drug	PEI	Tebu bio	24765-2	1 µg/µl
Chemical compound, drug	Penicillin-Streptomycin	Thermo Fisher Scientific	15140122	200 U/ml
Chemical compound, drug	ProLong Gold Antifade Reagent	Thermo Fsher Scientific	P36930	Pure
Chemical compound, drug	TransDux	System Biosciences	LV850A-1	1×
Chemical compound, drug	Triton X-100	Euromedex	2000C	1%
Chemical compound, drug	TrypLE	Gibco	12605	0.3×
Chemical compound, drug	TSA Plus Cyanine-3	Akoya Biosciences	NEL744001KT	1/750
Chemical compound, drug	TSA Plus Cyanine-5	Akoya Biosciences	NEL741001KT	1/750
Chemical compound, drug	TSA Plus Fluorescein	Akoya Biosciences	NEL766001KT	1/750
Chemical compound, drug	UEA	Vector Laboratories	RL-1062	1/50
Chemical compound, drug	Verteporfin	MilliporeSigma	SML0534	5–10 μM
Chemical compound, drug	Y27632	MilliporeSigma	Y0503	10 μM
Commercial assay or kit	EdU click-it kit	Thermo Fisher Scientific	C10340	
Commercial assay or kit	RNAscope Multiplex Fluorescent Detection Kit v2	ACD	323110	
Commercial assay or kit	RNAscope Probe Hs-LGR5-C3	ACD	311021-C3	
Commercial assay or kit	RNAscope Probe Hs-THBS1-C2	ACD	426581-C2	
Commercial assay or kit	RNAscope Probe Mm-CTGF	ACD	314541	
Commercial assay or kit	RNAscope Probe Mm-Lgr5	ACD	312171	
Commercial assay or kit	RNAscope Probe Mm-Thbs1-C3	ACD	57891-C3	
Gene (*H. sapiens*)	*Lgr5*	Ensembl	ENSG00000139292	
Gene (*H. sapiens*)	*Thbs1*	Ensembl	ENSG00000137801	
Gene (*H. sapiens*)	*Yap1*	Ensembl	ENSG00000137693	
Gene (*M. musculus*)	*Cp*	Ensembl	ENSMUSG00000003617	
Gene (*M. musculus*)	*Ctgf (CCN2*)	Ensembl	ENSMUSG00000019997	
Gene (*M. musculus*)	*Hdgf*	Ensembl	ENSMUSG00000004897	
Gene (*M. musculus*)	*Lgals-3*	Ensembl	ENSMUSG00000050335	
Gene (*M. musculus*)	*Lgals-3bp*	Ensembl	ENSMUSG00000033880	
Gene (*M. musculus*)	*Lgr5*	Ensembl	ENSMUSG00000020140	
Gene (*M. musculus*)	*Tead4*	Ensembl	ENSMUSG00000030353	
Gene (*M. musculus*)	*Thbs1*	Ensembl	ENSMUSG00000040152	
Gene (*M. musculus*)	*Ttr*	Ensembl	ENSMUSG00000061808	
Gene (*M. musculus*)	*Yap1*	Ensembl	ENSMUSG00000053110	
Other	Amicon Ultra Centrifugal Filters	MilliporeSigma	UFC910024	Filters used to concentrate the viral preparations
Peptide, recombinant protein	mEGF	Thermo Fisher Scientific	315-09	(50 ng/ml)
Peptide, recombinant protein	mNoggin	PeproTech	250-38	(100 ng/ml)
Peptide, recombinant protein	mRspo1	PeproTech	3474-RS	(500 ng/ml)
Peptide, recombinant protein	rmTHBS1	R&D Systems	7859-TH-050	(1–5 μg/ml)
Peptide, recombinant protein	Wnt3A	R&D Systems	1324-WN-002	(5 ng/ml)
Sequence-based reagent	STead4F	Eurofins Genomics	This paper	CTCTAACAGG TCCAACGGGC
Sequence-based reagent	STead4R	Eurofins Genomics	This paper	CAGCTCAGAC AGGCTCCTTAC
Sequence-based reagent	SThbs1F	Eurofins Genomics	This paper	GCGGGAGGTT TACCTGTGTG
Sequence-based reagent	SThbs1R	Eurofins Genomics	This paper	CCTCTTTAAAA GGTCCTGGGCT
Sequence-based reagent	SYap1F	Eurofins Genomics	This paper	GCCGCATGG GCACGGTCT
Sequence-based reagent	SYap1R	Eurofins Genomics	This paper	TGCGGGCG CGCGTCGC
Sequence-based reagent	Tead4-2 sgRNA	Eurofins Genomics	This paper	CCCATCGACA ATGATGCAGA
Sequence-based reagent	Thbs 1-1 sgRNA	Eurofins Genomics	This paper	CGGGGCTCA GTAACCCGGAG
Sequence-based reagent	Yap1-1 sgRNA	Eurofins Genomics	This paper	AGTCGGTCTC CGAGTCCCCG
Software, algorithm	ApE	https://jorgensen.biology.utah.edu/		v2.0.61
Software, algorithm	clusterProfiler	R package		v3.14.3
Software, algorithm	edgeR	PMID:19910308		v3.25.9
Software, algorithm	Fiji	https://imagej.net/		v1.53c
Software, algorithm	GSEA	https://gsea-msigdb.org/		v4.0.3
Software, algorithm	Ilastik	https://www.ilastik.org/		v1.3.2
Software, algorithm	Limma	PMID:25605792		
Software, algorithm	msigdbr	R package		v7.1.1
Software, algorithm	PerformanceAnalytics	R package		v2.0.4
Software, algorithm	STAR mapper	PMID:23104886		v2.5.3a
Software, algorithm	Thermo Scientific Proteome Discoverer	Thermo Fisher Scientific		v2.1
Software, algorithm	UniProt-GOA Mouse			v.20181203
Software, algorithm	Xcalibur	Thermo Fisher	OPTON-30965	v3.0
Strain, strain background (*M. musculus*)	*Apc* ^1638N^	PMID:8090754	MGI:1857951	
Strain, strain background (*M. musculus*)	*Apc*Δ14	PMID:15563600	MGI:3521822	
Strain, strain background (*M. musculus*)	C57BL/6	Charles Rivers	C57BL/6NCrl	Strain maintained in Institut Curie Mouse Facility
Strain, strain background (*M. musculus*)	Lgr5-GFP	PMID:17934449	MGI:3833921	
Strain, strain background (*M. musculus*)	LifeAct-GFP	PMID:18536722	MGI:6335778	
Strain, strain background (*M. musculus*)	R26CreERT2	PMID:17251932	MGI:3790674	
Strain, strain background (*M. musculus*)	R26-LSL-Cas9-GFP	PMID:25263330	MGI:25263330	
Strain, strain background (*M. musculus*)	R26mTmG	PMID:17868096	MGI:3722404	
Transfected construct	Lenti-7TG	Addgene	24314	
Transfected construct	LentiCas9Blast	Addgene	52962	
Transfected construct	Lenti-CRISPRv2	Addgene	82416	
Transfected construct	Lenti-sgRNA-GFP	Addgene	65656	
Transfected construct	Lenti-sgRNA-mTomato	This paper		Derived from Lenti-sgRNA-GFP
Transfected construct	LentiThbs1-FLAG	Origene	MR211744L3V	
Transfected construct	pMD2.G	Addgene	12259	
Transfected construct	psPAX2	Addgene	12260	

## References

[bib1] Azzolin L, Panciera T, Soligo S, Enzo E, Bicciato S, Dupont S, Bresolin S, Frasson C, Basso G, Guzzardo V, Fassina A, Cordenonsi M, Piccolo S (2014). YAP/TAZ incorporation in the β-catenin destruction complex orchestrates the Wnt response. Cell.

[bib2] Balkwill FR, Capasso M, Hagemann T (2012). The tumor microenvironment at a glance. Journal of Cell Science.

[bib3] Barker N, van Es JH, Kuipers J, Kujala P, van den Born M, Cozijnsen M, Haegebarth A, Korving J, Begthel H, Peters PJ, Clevers H (2007). Identification of stem cells in small intestine and colon by marker gene Lgr5. Nature.

[bib4] Barker N, Ridgway RA, van Es JH, van de Wetering M, Begthel H, van den Born M, Danenberg E, Clarke AR, Sansom OJ, Clevers H (2009). Crypt stem cells as the cells-of-origin of intestinal cancer. Nature.

[bib5] Barry ER, Morikawa T, Butler BL, Shrestha K, de la Rosa R, Yan KS, Fuchs CS, Magness ST, Smits R, Ogino S, Kuo CJ, Camargo FD (2013). Restriction of intestinal stem cell expansion and the regenerative response by YAP. Nature.

[bib6] Benjamini Y, Hochberg Y (1995). Controlling the False Discovery Rate: A Practical and Powerful Approach to Multiple Testing. Journal of the Royal Statistical Society.

[bib7] Brugmann SA, Goodnough LH, Gregorieff A, Leucht P, ten Berge D, Fuerer C, Clevers H, Nusse R, Helms JA (2007). Wnt signaling mediates regional specification in the vertebrate face. Development (Cambridge, England).

[bib8] Cai J, Maitra A, Anders RA, Taketo MM, Pan D (2015). β-Catenin destruction complex-independent regulation of Hippo-YAP signaling by APC in intestinal tumorigenesis. Genes & Development.

[bib9] Cheung P, Xiol J, Dill MT, Yuan W-C, Panero R, Roper J, Osorio FG, Maglic D, Li Q, Gurung B, Calogero RA, Yilmaz ÖH, Mao J, Camargo FD (2020). Regenerative Reprogramming of the Intestinal Stem Cell State via Hippo Signaling Suppresses Metastatic Colorectal Cancer. Cell Stem Cell.

[bib10] Cleary AS, Leonard TL, Gestl SA, Gunther EJ (2014). Tumour cell heterogeneity maintained by cooperating subclones in Wnt-driven mammary cancers. Nature.

[bib11] Colnot S, Decaens T, Niwa-Kawakita M, Godard C, Hamard G, Kahn A, Giovannini M, Perret C (2004). Liver-targeted disruption of Apc in mice activates beta-catenin signaling and leads to hepatocellular carcinomas. PNAS.

[bib12] Dobin A, Davis CA, Schlesinger F, Drenkow J, Zaleski C, Jha S, Batut P, Chaisson M, Gingeras TR (2013). STAR: ultrafast universal RNA-seq aligner. Bioinformatics (Oxford, England).

[bib13] Drost J, van Jaarsveld RH, Ponsioen B, Zimberlin C, van Boxtel R, Buijs A, Sachs N, Overmeer RM, Offerhaus GJ, Begthel H, Korving J, van de Wetering M, Schwank G, Logtenberg M, Cuppen E, Snippert HJ, Medema JP, Kops GJPL, Clevers H (2015). Sequential cancer mutations in cultured human intestinal stem cells. Nature.

[bib14] el Marjou F, Janssen K-P, Chang BH-J, Li M, Hindie V, Chan L, Louvard D, Chambon P, Metzger D, Robine S (2004). Tissue-specific and inducible Cre-mediated recombination in the gut epithelium. Genesis (New York, N.Y.

[bib15] Flanagan DJ, Pentinmikko N, Luopajärvi K, Willis NJ, Gilroy K, Raven AP, Mcgarry L, Englund JI, Webb AT, Scharaw S, Nasreddin N, Hodder MC, Ridgway RA, Minnee E, Sphyris N, Gilchrist E, Najumudeen AK, Romagnolo B, Perret C, Williams AC, Clevers H, Nummela P, Lähde M, Alitalo K, Hietakangas V, Hedley A, Clark W, Nixon C, Kirschner K, Jones EY, Ristimäki A, Leedham SJ, Fish PV, Vincent J-P, Katajisto P, Sansom OJ (2021). NOTUM from Apc-mutant cells biases clonal competition to initiate cancer. Nature.

[bib16] Fodde R, Edelmann W, Yang K, van Leeuwen C, Carlson C, Renault B, Breukel C, Alt E, Lipkin M, Khan PM (1994). A targeted chain-termination mutation in the mouse Apc gene results in multiple intestinal tumors. PNAS.

[bib17] Germann M, Xu H, Malaterre J, Sampurno S, Huyghe M, Cheasley D, Fre S, Ramsay RG (2014). Tripartite interactions between Wnt signaling, Notch and Myb for stem/progenitor cell functions during intestinal tumorigenesis. Stem Cell Research.

[bib18] Gregorieff A, Liu Y, Inanlou MR, Khomchuk Y, Wrana JL (2015). Yap-dependent reprogramming of Lgr5(+) stem cells drives intestinal regeneration and cancer. Nature.

[bib19] Guillermin O, Angelis N, Sidor CM, Ridgway R, Baulies A, Kucharska A, Antas P, Rose MR, Cordero J, Sansom O, Li VSW, Thompson BJ (2021). Wnt and Src signals converge on YAP-TEAD to drive intestinal regeneration. The EMBO Journal.

[bib20] Gutierrez LS, Suckow M, Lawler J, Ploplis VA, Castellino FJ (2003). Thrombospondin 1--a regulator of adenoma growth and carcinoma progression in the APC(Min/+) mouse model. Carcinogenesis.

[bib21] Hanahan D, Coussens LM (2012). Accessories to the crime: functions of cells recruited to the tumor microenvironment. Cancer Cell.

[bib22] Jacquemin G (2022a). Software Heritage.

[bib23] Jacquemin G (2022b). Software Heritage.

[bib24] Jardé T, Evans RJ, McQuillan KL, Parry L, Feng GJ, Alvares B, Clarke AR, Dale TC (2013). In vivo and in vitro models for the therapeutic targeting of Wnt signaling using a Tet-OΔN89β-catenin system. Oncogene.

[bib25] Krotenberg Garcia A, Fumagalli A, Le HQ, Jackstadt R, Lannagan TRM, Sansom OJ, van Rheenen J, Suijkerbuijk SJE (2021). Active elimination of intestinal cells drives oncogenic growth in organoids. Cell Reports.

[bib26] Liu-Chittenden Y, Huang B, Shim JS, Chen Q, Lee SJ, Anders RA, Liu JO, Pan D (2012). Genetic and pharmacological disruption of the TEAD-YAP complex suppresses the oncogenic activity of YAP. Genes & Development.

[bib27] Lopez-Dee ZP, Chittur SV, Patel H, Chinikaylo A, Lippert B, Patel B, Lawler J, Gutierrez LS (2015). Thrombospondin-1 in a Murine Model of Colorectal Carcinogenesis. PLOS ONE.

[bib28] Marusyk A, Tabassum DP, Altrock PM, Almendro V, Michor F, Polyak K (2014). Non-cell-autonomous driving of tumour growth supports sub-clonal heterogeneity. Nature.

[bib29] Merlos-Suárez A, Barriga FM, Jung P, Iglesias M, Céspedes MV, Rossell D, Sevillano M, Hernando-Momblona X, da Silva-Diz V, Muñoz P, Clevers H, Sancho E, Mangues R, Batlle E (2011). The intestinal stem cell signature identifies colorectal cancer stem cells and predicts disease relapse. Cell Stem Cell.

[bib30] Mourao L, Jacquemin G, Huyghe M, Nawrocki WJ, Menssouri N, Servant N, Fre S (2019). Lineage tracing of Notch1-expressing cells in intestinal tumours reveals a distinct population of cancer stem cells. Scientific Reports.

[bib31] Muzumdar MD, Tasic B, Miyamichi K, Li L, Luo L (2007). A global double-fluorescent Cre reporter mouse. Genesis (New York, N.Y.

[bib32] Ombrato L, Nolan E, Kurelac I, Mavousian A, Bridgeman VL, Heinze I, Chakravarty P, Horswell S, Gonzalez-Gualda E, Matacchione G, Weston A, Kirkpatrick J, Husain E, Speirs V, Collinson L, Ori A, Lee J-H, Malanchi I (2019). Metastatic-niche labelling reveals parenchymal cells with stem features. Nature.

[bib33] Ong SE, Blagoev B, Kratchmarova I, Kristensen DB, Steen H, Pandey A, Mann M (2002). Stable isotope labeling by amino acids in cell culture, SILAC, as a simple and accurate approach to expression proteomics. Molecular & Cellular Proteomics.

[bib34] Onuma K, Ochiai M, Orihashi K, Takahashi M, Imai T, Nakagama H, Hippo Y (2013). Genetic reconstitution of tumorigenesis in primary intestinal cells. PNAS.

[bib35] Perez-Riverol Y, Csordas A, Bai J, Bernal-Llinares M, Hewapathirana S, Kundu DJ, Inuganti A, Griss J, Mayer G, Eisenacher M, Pérez E, Uszkoreit J, Pfeuffer J, Sachsenberg T, Yilmaz S, Tiwary S, Cox J, Audain E, Walzer M, Jarnuczak AF, Ternent T, Brazma A, Vizcaíno JA (2019). The PRIDE database and related tools and resources in 2019: improving support for quantification data. Nucleic Acids Research.

[bib36] Platt RJ, Chen S, Zhou Y, Yim MJ, Swiech L, Kempton HR, Dahlman JE, Parnas O, Eisenhaure TM, Jovanovic M, Graham DB, Jhunjhunwala S, Heidenreich M, Xavier RJ, Langer R, Anderson DG, Hacohen N, Regev A, Feng G, Sharp PA, Zhang F (2014). CRISPR-Cas9 knockin mice for genome editing and cancer modeling. Cell.

[bib37] Poullet P, Carpentier S, Barillot E (2007). myProMS, a web server for management and validation of mass spectrometry-based proteomic data. Proteomics.

[bib38] Resovi A, Pinessi D, Chiorino G, Taraboletti G (2014). Current understanding of the thrombospondin-1 interactome. Matrix Biology.

[bib39] Riedl J, Crevenna AH, Kessenbrock K, Yu JH, Neukirchen D, Bista M, Bradke F, Jenne D, Holak TA, Werb Z, Sixt M, Wedlich-Soldner R (2008). Lifeact: a versatile marker to visualize F-actin. Nature Methods.

[bib40] Ring DB, Johnson KW, Henriksen EJ, Nuss JM, Goff D, Kinnick TR, Ma ST, Reeder JW, Samuels I, Slabiak T, Wagman AS, Hammond M-EW, Harrison SD (2003). Selective Glycogen Synthase Kinase 3 Inhibitors Potentiate Insulin Activation of Glucose Transport and Utilization In Vitro and In Vivo. Diabetes.

[bib41] Ritchie ME, Phipson B, Wu D, Hu Y, Law CW, Shi W, Smyth GK (2015). limma powers differential expression analyses for RNA-sequencing and microarray studies. Nucleic Acids Research.

[bib42] Robinson MD, McCarthy DJ, Smyth GK (2010). edgeR: a Bioconductor package for differential expression analysis of digital gene expression data. Bioinformatics (Oxford, England).

[bib43] Sanjana NE, Shalem O, Zhang F (2014). Improved vectors and genome-wide libraries for CRISPR screening. Nature Methods.

[bib44] Sato T, Vries RG, Snippert HJ, van de Wetering M, Barker N, Stange DE, van Es JH, Abo A, Kujala P, Peters PJ, Clevers H (2009). Single Lgr5 stem cells build crypt-villus structures in vitro without a mesenchymal niche. Nature.

[bib45] Sato T, Stange DE, Ferrante M, Vries RGJ, Van Es JH, Van den Brink S, Van Houdt WJ, Pronk A, Van Gorp J, Siersema PD, Clevers H (2011). Long-term expansion of epithelial organoids from human colon, adenoma, adenocarcinoma, and Barrett’s epithelium. Gastroenterology.

[bib46] Sato T, Clevers H (2013). Growing self-organizing mini-guts from a single intestinal stem cell: mechanism and applications. Science (New York, N.Y.).

[bib47] Schwank G, Koo B-K, Sasselli V, Dekkers JF, Heo I, Demircan T, Sasaki N, Boymans S, Cuppen E, van der Ent CK, Nieuwenhuis EES, Beekman JM, Clevers H (2013). Functional repair of CFTR by CRISPR/Cas9 in intestinal stem cell organoids of cystic fibrosis patients. Cell Stem Cell.

[bib48] Shalem O, Sanjana NE, Hartenian E, Shi X, Scott DA, Mikkelson T, Heckl D, Ebert BL, Root DE, Doench JG, Zhang F (2014). Genome-scale CRISPR-Cas9 knockout screening in human cells. Science (New York, N.Y.).

[bib49] Sid B, Langlois B, Sartelet H, Bellon G, Dedieu S, Martiny L (2008). Thrombospondin-1 enhances human thyroid carcinoma cell invasion through urokinase activity. The International Journal of Biochemistry & Cell Biology.

[bib50] Sun W, Duan T, Ye P, Chen K, Zhang G, Lai M, Zhang H (2018). TSVdb: a web-tool for TCGA splicing variants analysis. BMC Genomics.

[bib51] Tanaka T, Kohno H, Suzuki R, Yamada Y, Sugie S, Mori H (2003). A novel inflammation-related mouse colon carcinogenesis model induced by azoxymethane and dextran sodium sulfate. Cancer Science.

[bib52] Taniguchi K, Wu L-W, Grivennikov SI, de Jong PR, Lian I, Yu F-X, Wang K, Ho SB, Boland BS, Chang JT, Sandborn WJ, Hardiman G, Raz E, Maehara Y, Yoshimura A, Zucman-Rossi J, Guan K-L, Karin M (2015). A gp130-Src-YAP module links inflammation to epithelial regeneration. Nature.

[bib53] Taniguchi K, Moroishi T, de Jong PR, Krawczyk M, Grebbin BM, Luo H, Xu RH, Golob-Schwarzl N, Schweiger C, Wang K, Di Caro G, Feng Y, Fearon ER, Raz E, Kenner L, Farin HF, Guan KL, Haybaeck J, Datz C, Zhang K, Karin M (2017). YAP-IL-6ST autoregulatory loop activated on APC loss controls colonic tumorigenesis. PNAS.

[bib54] Teraoku H, Morine Y, Ikemoto T, Saito Y, Yamada S, Yoshikawa M, Takasu C, Higashijima J, Imura S, Shimada M (2016). Role of thrombospondin-1 expression in colorectal liver metastasis and its molecular mechanism. Journal of Hepato-Biliary-Pancreatic Sciences.

[bib55] Tuszynski GP, Gasic TB, Rothman VL, Knudsen KA, Gasic GJ (1987). Thrombospondin, a potentiator of tumor cell metastasis. Cancer Research.

[bib56] van Neerven SM, de Groot NE, Nijman LE, Scicluna BP, van Driel MS, Lecca MC, Warmerdam DO, Kakkar V, Moreno LF, Vieira Braga FA, Sanches DR, Ramesh P, Ten Hoorn S, Aelvoet AS, van Boxel MF, Koens L, Krawczyk PM, Koster J, Dekker E, Medema JP, Winton DJ, Bijlsma MF, Morrissey E, Léveillé N, Vermeulen L (2021). Apc-mutant cells act as supercompetitors in intestinal tumour initiation. Nature.

[bib57] Ventura A, Kirsch DG, McLaughlin ME, Tuveson DA, Grimm J, Lintault L, Newman J, Reczek EE, Weissleder R, Jacks T (2007). Restoration of p53 function leads to tumour regression in vivo. Nature.

[bib58] Yamashiro Y, Thang BQ, Ramirez K, Shin SJ, Kohata T, Ohata S, Nguyen TAV, Ohtsuki S, Nagayama K, Yanagisawa H (2019). Matrix Mechanotransduction Mediated by Thrombospondin-1/Integrin/YAP Signaling Pathway in the Remodeling of Blood Vessels. bioRxiv.

[bib59] Yui S, Azzolin L, Maimets M, Pedersen MT, Fordham RP, Hansen SL, Larsen HL, Guiu J, Alves MRP, Rundsten CF, Johansen JV, Li Y, Madsen CD, Nakamura T, Watanabe M, Nielsen OH, Schweiger PJ, Piccolo S, Jensen KB (2018). YAP/TAZ-Dependent Reprogramming of Colonic Epithelium Links ECM Remodeling to Tissue Regeneration. Cell Stem Cell.

[bib60] Yum MK, Han S, Fink J, Wu S-HS, Dabrowska C, Trendafilova T, Mustata R, Chatzeli L, Azzarelli R, Pshenichnaya I, Lee E, England F, Kim JK, Stange DE, Philpott A, Lee J-H, Koo B-K, Simons BD (2021). Tracing oncogene-driven remodelling of the intestinal stem cell niche. Nature.

[bib61] Zanconato F, Cordenonsi M, Piccolo S (2016). YAP/TAZ at the Roots of Cancer. Cancer Cell.

