# Supplementary figures and images for: Paracrine signalling between intestinal epithelial and tumour cells induces a regenerative programme
Source: eLife. 2022 May 11;11:e76541. doi: 10.7554/eLife.76541 (PMC9094746; doi:10.7554/eLife.76541)

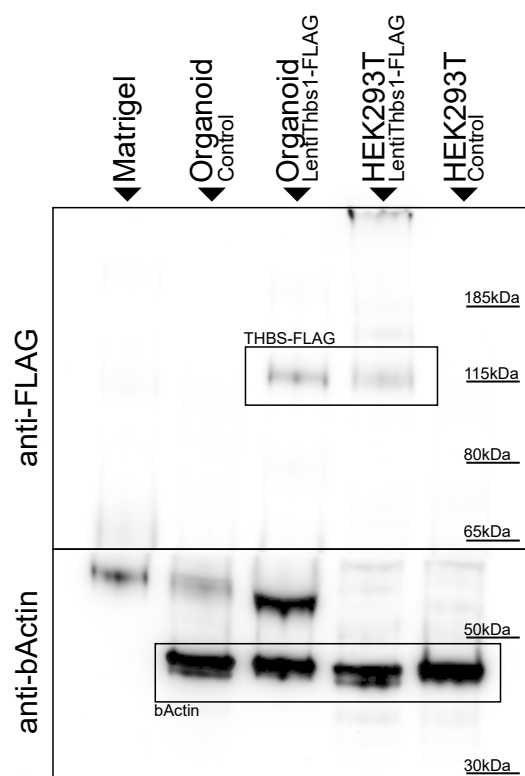

Supplement: Figure 2—figure supplement 1—source data 1. [file elife-76541-fig2-figsupp1-data1.pdf]

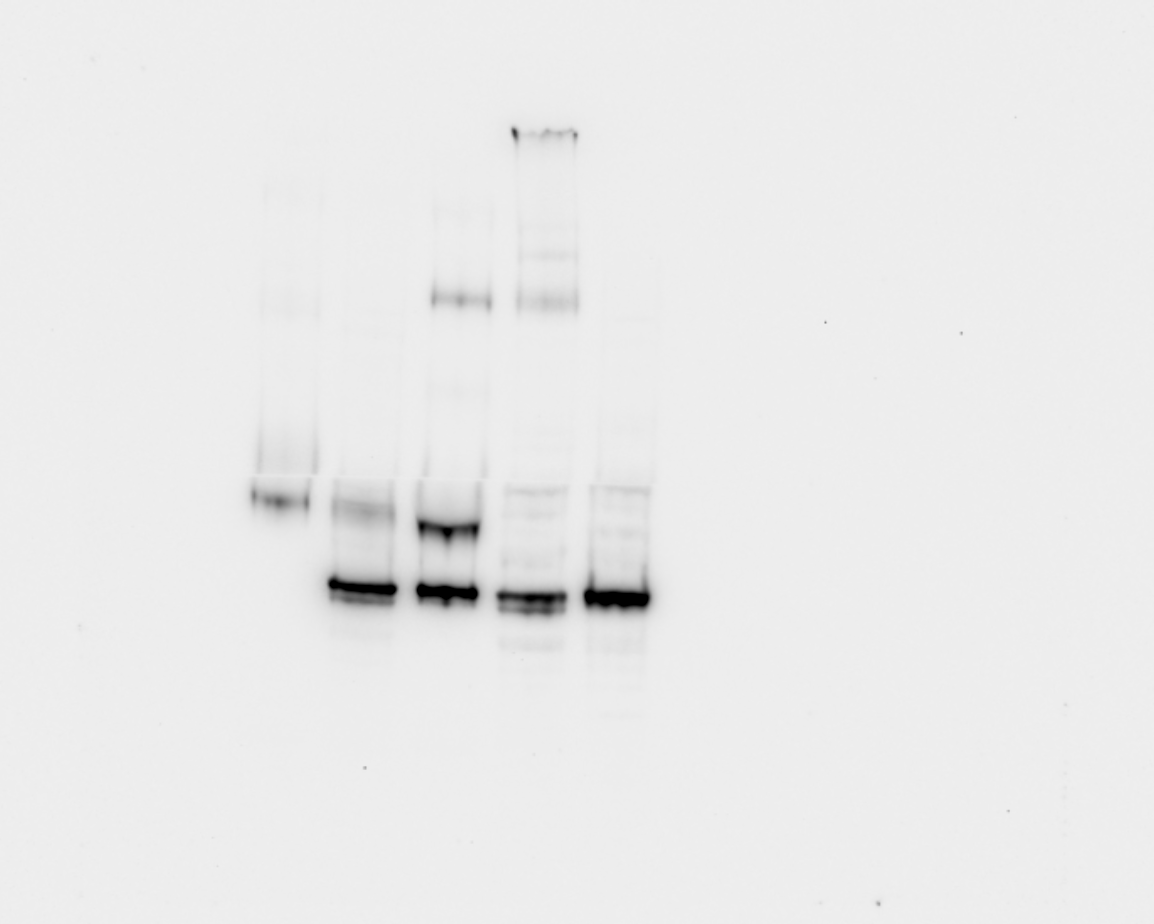

Supplement: Figure 2—figure supplement 1—source data 2. [file elife-76541-fig2-figsupp1-data2.zip › gui 2021-10-15 16h35m07s(Chemiluminescence).tif]
